# Task-dependent cortical responses to peripheral and central neuromodulation during water and saliva swallowing after stroke: an exploratory fNIRS pilot study

**DOI:** 10.3389/fnhum.2026.1907700

**Published:** 2026-08-24

**Authors:** Yuru Song, Licong Gao, Baohu Liu, Guangjian Shao, Ming Qi, Shuai Yang, Meijie Sun, Congcong Huo, Jianqi Song, Xiaoxia Du

**Affiliations:** 1 Beijing Daxing District Hospital of Integrated Chinese and Western Medicine, Beijing, China; 2 Wangjing Hospital, China Academy of Chinese Medical Science, Beijing, China; 3 Key Laboratory of Rehabilitation Aids Technology and System of the Ministry of Civil Affairs, National Research Center for Rehabilitation Technical Aids, Beijing, China; 4 School of Rehabilitation Medicine, Capital Medical University, Beijing, China; 5 Department of Neurorehabilitation, China Rehabilitation Research Center, Beijing, China

**Keywords:** fNIRS, hemispheric lateralization, neuromodulation, post-stroke dysphagia, swallowing tasks

## Abstract

**Background:**

Post-stroke dysphagia is a common and serious complication. Neurorehabilitation strategies include peripheral neuromuscular electrical stimulation (NMES) and central transcranial direct current stimulation (tDCS), which operate via distinct bottom-up and top-down mechanisms, respectively. However, their differential effects on cortical hemodynamic responses during functionally distinct swallowing tasks remain poorly understood. This pilot study aimed to investigate the immediate modulatory effects of NMES and tDCS on cortical hemodynamics during different swallowing tasks in post-stroke patients using functional near-infrared spectroscopy (fNIRS).

**Methods:**

Eleven patients with post-stroke dysphagia participated in this exploratory, within-subject, repeated-measures pilot study. A minimum 24-h washout period was maintained between sessions, and the order of intervention conditions was randomized using a computer-generated sequence. Cortical hemodynamic responses, measured by changes in oxygenated hemoglobin (ΔHbO₂), were monitored using a 56-channel fNIRS system. Each participant performed both water swallowing and saliva swallowing tasks under three randomized conditions: a no-stimulation baseline, peripheral stimulation (NMES applied to suprahyoid muscles), and central stimulation (bilateral anodal tDCS over the primary motor cortex). Task-related cortical hemodynamic responses and hemispheric lateralization were analyzed for key regions of interest.

**Results:**

The primary analysis identified significant Stimulation × Task interactions in the bilateral frontopolar cortex (FPC), including the ipsilesional FPC, *F*(2, 20) = 8.44, raw *p* = 0.002, q = 0.031, partial η^2^ = 0.458, and the contralesional FPC, *F*(2, 20) = 7.42, raw *p* = 0.004, q = 0.031, partial η^2^ = 0.426. Bonferroni-adjusted simple-effects analyses showed that, under tDCS, water swallowing elicited greater FPC responses than saliva swallowing in both the ipsilesional (p_adj = 0.020) and contralesional (p_adj = 0.008) FPC. During water swallowing, tDCS produced greater contralesional FPC responses than no stimulation (p_adj = 0.006), whereas during saliva swallowing, no stimulation produced greater bilateral FPC responses than tDCS (I-FPC: p_adj = 0.008; C-FPC: p_adj = 0.034) and greater ipsilesional FPC responses than NMES (p_adj = 0.043). Exploratory paired comparisons, with FDR correction applied within each comparison family, further showed that under the no-stimulation condition, saliva swallowing elicited greater bilateral FPC responses than water swallowing (I-FPC: q = 0.018; C-FPC: q = 0.024). Under NMES, water swallowing elicited greater ipsilesional FPC responses than saliva swallowing (q = 0.002). During saliva swallowing, tDCS was associated with greater ipsilesional SMA/PMC responses than NMES (q = 0.014).

**Conclusion:**

Acute cortical hemodynamic responses varied according to the combination of stimulation condition and swallowing task, with the strongest evidence arising from the bilateral FPC Stimulation × Task interaction. These findings indicate task-dependent prefrontal hemodynamic responses but do not establish stimulation-specific efficacy, hemispheric reorganization, neuroplasticity, or therapeutic superiority. Larger sham-controlled studies are required to determine the reproducibility and clinical relevance of these acute fNIRS findings.

**Clinical trial registration:**

The trial is registered at the International Traditional Medicine Clinical Trial Registry (ITMCTR), Available at: https://itmctr.ccebtcm.org.cn/en/index.html, Identifier ITMCTR2025001814.

## 1 Introduction

Swallowing is a complex sensorimotor process requiring coordinated interactions among cortical, subcortical, brainstem, and peripheral neuromuscular systems (Wei et al., 2024). Research indicates that the central reflex center for swallowing is situated in the brainstem, particularly in the Dorsal Swallowing Group (DSG) and Ventral Swallowing Group (VSG) of the medulla oblongata (Murakawa and Satoh, 2024). The initiation of swallowing and the coordination of oral and tongue movements are primarily influenced by brain areas located in the cerebral cortex, such as the sensorimotor cortex, precentral gyrus, insular cortex, and frontal lobe, as well as subcortical structures like the basal ganglia and thalamus. These regions play key roles in voluntary initiation, sensory integration, and attentional regulation of swallowing. Swallowing initiation is a reflexive and autonomously controlled action that heavily depends on sensory feedback and sensorimotor integration (Rofes et al., 2017; Cabib et al., 2020). It is primarily regulated by the coordinated control of the cerebral cortex and subcortical regions (Mélotte et al., 2023). In stroke patients, damage to supratentorial swallowing-related networks or their descending projections results in severe swallowing dysfunction, leading to complications such as aspiration pneumonia and malnutrition (Wilmskoetter et al., 2020). Therefore, developing effective swallowing rehabilitation techniques is clinically significant.

In recent years, there have been two main strategies for neurorehabilitation in swallowing disorders: peripheral approaches involving neuromuscular electrical stimulation (NMES) or pharyngeal electrical stimulation to enhance sensory input to the central nervous system, and central nervous system modulation techniques such as transcranial direct current stimulation (tDCS) or transcranial magnetic stimulation (TMS) targeting the pharyngeal motor cortex to facilitate recovery of brain swallowing function (Clavé et al., 2020). NMES typically targets the suprahyoid muscles, which are crucial for the pharyngeal phase of swallowing, responsible for elevating the larynx and protecting the airway (Kitamura et al., 2024). Research indicates that NMES not only prevents disuse atrophy by inducing muscle contractions but also reduces the risk of aspiration by enhancing sensory feedback (bottom-up) (Tarihci Cakmak et al., 2023; Oh et al., 2020). tDCS, a non-invasive technique, modulates the resting membrane potential of cortical neurons by applying weak direct current to specific areas of the scalp (top-down) (Zhu and Gu, 2022). Given that swallowing is controlled by both brain hemispheres (Daniels et al., 2006), utilizing tDCS to promote neuroplasticity in the healthy or affected hemisphere has been shown to aid in swallowing function recovery (Tu et al., 2021).

Water swallowing and saliva swallowing are common oropharyngeal behaviors in daily life. However, they exhibit significant differences in activation patterns in the cerebral cortex, reflecting varying complexities in motor control, sensory feedback, and cognitive involvement (Lin, 2019). In the present cued paradigm, saliva swallowing represents a more internally generated, volitional swallowing task that relies on central pattern generators in the brainstem and requires involvement of higher cortical centers, such as the inferior frontal gyrus, motor prefrontal area, and somatosensory cortex, for motor planning and initiation (Huang et al., 2023; Knollhoff et al., 2022; Kober and Wood, 2018). In contrast, water swallowing is a reflexive swallowing task triggered by sensory inputs (volume, pressure, temperature) in the oropharynx, leading to a cascade of muscle responses in the swallowing muscles through various pathways involving cortical and brainstem pathways, activating the cortical sensorimotor network, including bilateral inferior frontal gyrus and left precentral gyrus, to reflexively modulate motor output throughout the swallowing sequence (Steele and Miller, 2010; Hashimoto et al., 2021; Gu et al., 2024).

In current clinical research, there is a predominant focus on the efficacy of single stimulation techniques, while there is limited exploration into how NMES and tDCS, two different mechanisms of stimulation, modulate cortical activity differently during various swallowing tasks (reflexive vs. voluntary). NMES primarily induces peripheral effects by directly stimulating peripheral nerves and muscles, while tDCS modulates cortical neuron excitability, exerting central nervous system regulation. These two techniques target distinct sites and mechanisms. Reflexive swallowing in the neural regulation of swallowing is mainly controlled by the brainstem swallowing center and is characterized by automation. In contrast, voluntary swallowing requires higher cognitive involvement of the cortical areas, involving complex cortical–subcortical network interactions. Therefore, these stimulation techniques may exhibit different neurobiological effects when modulating different types of swallowing tasks. Currently, understanding of the differential modulation mechanisms of cortical activity by these two stimulation methods during various swallowing tasks remains insufficient, highlighting the crucial importance of elucidating this issue for optimizing clinical rehabilitation protocols.

In recent years, functional near-infrared spectroscopy (fNIRS) has emerged as a crucial tool for investigating swallowing-related brain function, owing to its non-invasiveness, portability, and good tolerance to motion artifacts (Delorme et al., 2019; Zimmermann et al., 2013). In comparison to fMRI, fNIRS allows participants to perform swallowing tasks in a relatively natural seated position, offering higher ecological validity and effectively mitigating interference from muscle movements in the head and neck during swallowing (Knollhoff et al., 2022; Wen et al., 2023; Chua and Chan, 2025). Therefore, this technique is particularly suitable for real-time assessment of cortical hemodynamic responses in dysphagia patients performing specific tasks. In this study, functional near-infrared spectroscopy (fNIRS) was utilized to observe the cortical hemodynamic response patterns of post-stroke dysphagia patients during water and saliva swallowing tasks under three different conditions (no stimulation, NMES, tDCS). The study aims to compare the immediate modulatory effects of different interventions on cortical excitability and hemispheric lateralization patterns, explore the task-stimulation matching hypothesis, and provide neuroimaging evidence for tailoring precise rehabilitation strategies based on specific swallowing tasks of individual patients in clinical practice.

## 2 Materials and methods

### 2.1 Participants

A total of 15 stroke patients with dysphagia admitted to the Department of Rehabilitation Medicine at a hospital specializing in integrated traditional Chinese and Western medicine in Daxing District, Beijing, between June 2025 and December 2025 were recruited for this study. Given the exploratory nature of this investigation, the sample size was determined based on feasibility considerations rather than a formal *a priori* power calculation. The study population included patients with heterogeneous lesion locations spanning thalamic, basal ganglia, frontoparietal, and periventricular regions across both hemispheres and both ischemic and hemorrhagic stroke subtypes (see Table 1 for details). All participants were confirmed as right-handed using the Edinburgh Handedness Inventory (Edlin et al., 2015). Inclusion criteria: (1) Unilateral hemisphere stroke confirmed by clinical symptoms, signs, and imaging examinations (CT and/or MRI); (2) Clinical diagnosis of dysphagia based on Functional Oral Intake Scale (FOIS≤6) and Modified Water Swallowing Test (MWST ≥ level 3); (3) Stable vital signs and a disease duration of ≥2 weeks; (4) Age between 18 and 85 years old; and (5) Ability to understand and cooperate with the study procedures. Exclusion criteria: (1) Severe cognitive impairment or aphasia leading to inability to follow instructions; (2) Concurrent severe cardiopulmonary dysfunction, epilepsy, or local skin lesions; (3) Coexisting conditions that may cause dysphagia (e.g., head and neck tumors, history of radiotherapy or chemotherapy, tongue muscle atrophy, myasthenia gravis, multiple sclerosis, etc.); and (4) Presence of metal implants in the scalp or contraindications to tDCS such as cardiac pacemakers. Patient demographics, including gender, age, duration of illness, lesion location, and stroke type, as well as swallowing function, were assessed by a professional therapist (refer to Table 1). The MWST and FOIS scores presented in Table 1 were obtained at study enrollment as baseline measures to characterize the participants’ swallowing status and assess eligibility. These enrollment assessments were distinct from the session-specific pre- and post-intervention KWDT and FOIS assessments used as clinical outcome measures, as described in Section 2.3.

**Table 1:** Baseline characteristics of enrolled participants at study enrollment.

No.	Age	Sex	Time after stroke (months)	Stroke type and Lesion location	Swallowing evaluation		Included in final fNIRS analysis
					MWST	FOIS	Yes/No
Pat 1	56	Male	0.5	Infarction_Right thalamus	4	3	Yes
Pat 2	56	Male	1	Infarction_Right centrum semiovale	5	1	Yes
Pat 3	62	Female	0.5	Infarction_Right frontal, temporal, and parietal lobes	4	2	Yes
Pat 4	58	Male	1	Infarction_Right Periventricular	4	2	Yes
Pat 5	85	Male	0.5	Hemorrhage_Right Occipitoparietal	3	4	Yes
Pat 6	53	Male	5	Hemorrhage_Left frontal, temporal, and parietal lobes	3	4	Yes
Pat 7	66	Female	2	Infarction_Left basal ganglia	3	4	Yes
Pat 8	79	Female	2	Infarction_Left basal ganglia	4	3	Yes
Pat 9	73	Male	2	Infarction_Left basal ganglia	3	4	Yes
Pat 10	83	Male	0.5	Infarction_Left basal ganglia	4	3	Yes
Pat 11	64	Female	1	Infarction_Left frontal, temporal, and parietal lobes	5	1	Yes
Pat 12	70	Male	2	Hemorrhage_Left basal ganglia	4	2	No
Pat 13	56	Male	0.5	Infarction_Left basal ganglia	4	3	No
Pat 14	62	Female	1	Hemorrhage_Right Occipitoparietal	3	4	No
Pat 15	78	Female	0.5	Infarction_Left basal ganglia	5	1	No

MWST, Modified Water Swallowing Test score; FOIS, Functional Oral Intake Scale score.

The research protocol followed the ethical guidelines outlined in the Helsinki Declaration and received approval from the hospital’s ethics committee (ethical approval number: DXZXYQ202405). The project was registered on the International Traditional Medicine Clinical Trials Registry platform (registration number: ITMCTR2025001814). All participants were fully informed about the research objectives and procedures and provided written consent prior to the study commencement.

### 2.2 Experimental design and data acquisition

**Figure 1:**
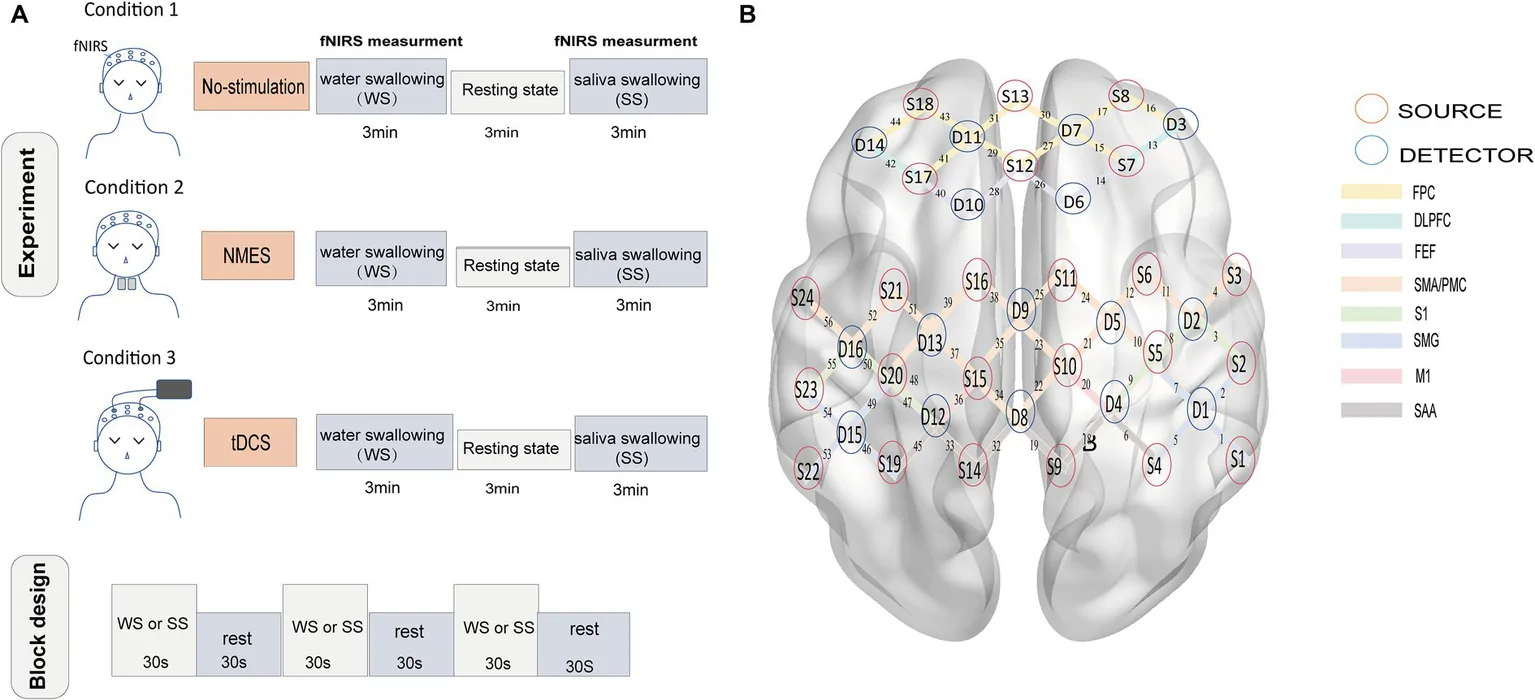
Experimental design and fNIRS acquisition. **(A)** Each participant completed the water swallowing and saliva swallowing tasks in three intervention conditions: No stimulation, NMES, and tDCS, assigned randomly. A minimum 24-h washout interval was used between stimulation sessions. Within each session, a 3-min rest interval separated the two swallowing tasks. **(B)** Schematic diagram of fNIRS probe configuration based on the International 10–20 electrode system, consisting of 24 light source probes and 16 detector probes forming 56 channels.

The research procedure, as illustrated in Figure 1A, employed a within-subject repeated measures design. Each participant completed three sessions at different times, being randomly allocated to three distinct intervention conditions, while concurrently executing swallowing tasks. The order of conditions was randomized using a computer-generated randomization sequence for each participant. A minimum washout period of 24 h was maintained between consecutive sessions to minimize potential carry-over effects.

#### 2.2.1 Intervention conditions

The three intervention conditions are as follows: (1) Condition 1: In the no-stimulation condition, participants performed the swallowing tasks without active stimulation. (2) Condition 2: Peripheral stimulation was delivered using a swallowing nerve and muscle electrical stimulator (YS1001P, Changzhou Siya Medical Equipment Co., Ltd., China). Surface electrodes were placed bilaterally over the submental/suprahyoid region, targeting the anterior belly of the digastric and adjacent suprahyoid musculature. Stimulation was delivered using the automatic-trigger function of the device’s single-pulse training mode. In this mode, individual stimulation pulses were delivered automatically according to the preset device program without manual triggering by the participant or therapist. The stimulation was not triggered by an electromyographic threshold or by the occurrence of an individual swallowing event. Based on the device settings used during the study, the stimulation intensity was 1.5 mA, with a pulse width of 400 ms and an inter-pulse interval of 5 s. NMES was administered during the 20-min intervention period. The swallowing task blocks were performed during this period; however, the timing of individual swallowing cues and swallowing events was not used to trigger stimulation and was not time-locked to individual stimulation pulses. Bilateral 5 × 5 cm surface electrodes were positioned over the suprahyoid region. (3) Condition 3: central stimulation based on tDCS was administered using a commercially available system (Starstim^®^ tES-EEG system), anodes were placed over bilateral M1 (C3 and C4), and the cathode was placed at FC6, with current intensities of 1 mA at C3 and C4, and -2 mA at FC6 (according to the 10–20 electroencephalogram system) (Nguyen et al., 2022; Suntrup-Krueger et al., 2018). Bilateral M1 was selected as the stimulation target as a study-specific design choice, considering the bilateral cortical involvement in swallowing-related motor control and previous evidence supporting the feasibility of bilateral stimulation of swallowing-related motor cortices after stroke (Ahn et al., 2017; Duan et al., 2025). The specific C3/C4 anodal-FC6 return montage used in the present study was not directly derived from these previous protocols. Accordingly, the electrode current densities were +0.04 mA/cm^2^ at each anode and −0.08 mA/cm^2^ at the FC6 return electrode, corresponding to an absolute current density of 0.08 mA/cm^2^ at FC6. Electrode impedance was checked before stimulation and continuously monitored throughout each session using the device software. The swallowing tasks were performed during the 20-min stimulation period (online tDCS), with fNIRS data acquired concurrently. A 20s ramp-up and ramp-down period is consistent with commonly used tDCS protocols.

#### 2.2.2 Swallowing tasks

Two swallowing tasks were used: (1) water swallowing task (WS), where researchers administer 5 mL of water into the mouth using a disposable plastic pipette; and (2) saliva swallowing task (SS), where participants perform voluntary swallowing actions based on verbal cues (without any physical inducement). For the saliva swallowing task, a standardized verbal cue (“Please swallow your saliva now”) was delivered by the same investigator throughout the study at a consistent volume and tone. Each participant completed both the WS and SS tasks under three distinct intervention conditions: No stimulation (control condition with no intervention), NMES, and tDCS. The order of these three intervention conditions was assigned randomly to each participant to minimize potential order effects and ensure the validity of the experimental design. The order of the three intervention conditions was randomized for each participant using a computer-generated sequence, with a minimum 24-h washout interval between consecutive sessions to minimize carry-over effects. Within each session, the WS and SS tasks were performed sequentially and were separated by a 3-min rest interval. For each swallowing task, participants completed three task-rest cycles. Each task block lasted 30 s and included three swallowing events, followed by a 30-s rest block. The task order was kept consistent across participants to ensure procedural standardization.

#### 2.2.3 fNIRS data acquisition

Participants were required to maintain a resting state, sitting comfortably, remaining awake with eyes open, and refraining from engaging in active cognitive processes before each intervention condition. Subsequently, perform swallowing tasks under three intervention conditions: no stimulation, NMES, and tDCS, while synchronously collecting fNIRS data.

A portable fNIRS imaging system (NirSmart, produced by HuiChuang Medical Equipment Co., Ltd., China) was used to monitor cerebral hemodynamic changes during swallowing in stroke patients. The system operates at 11 Hz sampling frequency with wavelengths of 850 nm and 730 nm. Based on the 10–20 electrode placement system, it comprises 24 light source probes and 16 detection probes (56 effective channels) with 3 cm probe spacing (Figure 1B). Spatial registration was performed using the NirScan localization system in conjunction with standard head modeling and 3D localization techniques to confirm the precise position of the probe on the scalp surface. The midpoint of each channel, determined by MNI and Talairach coordinates, served as the primary detection area for identifying corresponding brain regions. According to functional anatomical divisions, the region of interest (ROI) comprises a total of 16 cortical subregions bilaterally, including bilateral frontopolar cortex (FPC), frontal eye field (FEF), dorsolateral prefrontal cortex (DLPFC), primary motor cortex (M1), somatosensory association cortex (SAA), primary somatosensory cortex (S1), supplementary motor area/premotor cortex (SMA/PMC), and supramarginal gyrus (SMG).

#### 2.2.4 fNIRS data preprocessing and analysis

The analysis of fNIRS data was conducted utilizing MATLAB (MathWorks, Natick, Massachusetts, United States) with the HOMER2 software package (Huppert, 2016). First, converted the raw data into optical density values. Next, utilized the hmrMotionArtifactByChannel and hmrMotionCorrectionSpline algorithms from the HOMER2 fNIRS processing software package to identify and eliminate peak artifacts (hmrMotionArtifactByChannel parameters: STDEVthresh = 25.0, AMPthresh = 2.50, tMotion = 0.5 s, tMask = 1.0 s; hmrMotionCorrectionSpline parameter: *p* = 0.99). Because short-separation channels were unavailable, PCA-based correction was applied to attenuate superficial hemodynamic components (Zhang et al., 2005). Perform bandpass filtering on the fNIRS data (with a low cutoff frequency of 0.01 Hz and a high cutoff frequency of 0.1 Hz) to eliminate the influence of heartbeat, respiration, and low-frequency signal drift at various wavelengths (Yücel et al., 2021). The preprocessed optical-density signals were converted into changes in oxygenated and deoxygenated hemoglobin concentrations using the modified Beer–Lambert law. A fixed differential pathlength factor (DPF) of 6.0 was applied to both wavelengths (730 and 850 nm) as an approximation based on experimentally measured adult-head DPF values in the near-infrared range (Duncan et al., 1995; Zhao et al., 2002). Because DPF varies with wavelength, age, and measurement location, the use of a fixed DPF represents an approximation and was applied consistently across all participants and experimental conditions. The primary statistical analyses focused on ΔHbO_2_ because HbO_2_ has shown greater sensitivity to task-related cortical responses and higher test–retest reproducibility than HbR in several task-based fNIRS studies (Wagner et al., 2025; Yang et al., 2020; Plichta et al., 2006; Ferrari and Quaresima, 2012). However, this choice does not imply that HbO_2_ is universally superior to HbR across all paradigms. Finally, a block averaging analysis was performed (hmrBlockAvg, trange = [−2.0, 20.0] s relative to task onset) to estimate the hemodynamic response function (HRF) for each task. To facilitate group-level comparisons across patients with heterogeneous lesion laterality, data from participants with left-hemisphere lesions were spatially mirrored before statistical analysis so that the ipsilesional hemisphere was consistently represented on the right side. This normalization procedure has been widely adopted in stroke fNIRS studies to enable ROI-wise and hemispheric lateralization analyses across participants with different lesion sides (Wang et al., 2026). Participants whose fNIRS recordings remained dominated by motion artifacts after preprocessing and motion correction, preventing reliable estimation of the hemodynamic response, were excluded from further statistical analyses. Per-participant channel exclusion counts were recorded during fNIRS preprocessing. Among the 11 participants retained for final analysis, the number of excluded channels for each participant was less than 20% of the total 56 channels, indicating that sufficient channel coverage was preserved for ROI-level analysis.

To observe the laterality of cortical hemodynamic responses in the ipsilesional and contralesional hemispheres under different task conditions, calculate the laterality index (LI) as follows: LI= $\frac{\Delta \text{ipsilesional}\_\mathrm{HbO}-\Delta \text{contralesional}\_\mathrm{HbO}}{\Delta \text{ipsilesional}\_\mathrm{HbO}+\Delta \text{contralesional}\_\mathrm{HbO}}.$ Here, LI > 0 indicates stronger brain activity in the ipsilesional hemisphere, LI < 0 indicates stronger brain activity in the contralesional hemisphere, and LI close to 0 suggests balanced activity in both hemispheres.

### 2.3 Clinical outcome assessment

To evaluate the functional significance of stimulation-induced cortical changes, two clinical swallowing assessment tools, the Kubota Water Drinking Test (KWDT) and the Functional Oral Intake Scale (FOIS), were administered before and after each stimulation session. These session-specific assessments were conducted separately from the enrollment baseline assessments reported in Table 1. The MWST and FOIS values in Table 1 were obtained at study enrollment to characterize baseline swallowing status and determine eligibility, whereas the KWDT and FOIS assessments described here were repeated before and after each intervention session as clinical outcome measures. The KWDT requires participants to drink 30 mL of room-temperature water in a seated position and rates swallowing function on a 5-level scale (Level I: able to drink in one gulp without choking; Level II: able to drink in two or more gulps without choking; Level III: able to drink in one gulp with choking; Level IV: able to drink in two or more gulps with choking; Level V: frequent choking or inability to drink). The FOIS is a 7-level ordinal scale assessing the functional level of oral intake (Level 1: nothing by mouth; Level 7: total oral diet with no restrictions). Both assessments were conducted by trained speech-language pathologists who were blinded to the stimulation condition and were not involved in intervention delivery or randomization. The outcome assessors were not present during the stimulation sessions and had no access to the randomization sequence, session allocation, or stimulation records. Before the post-stimulation assessment, all stimulation equipment and electrodes were removed, thereby minimizing visual cues regarding the preceding intervention condition. Outcome assessors were blinded to the stimulation condition, whereas participants and personnel delivering the interventions were not blinded. The pre-stimulation assessment was performed within 10 min before the stimulation onset, and the post-stimulation assessment was conducted within 10 min after the completion of the fNIRS recording session.

### 2.4 Statistical analysis

**Figure 2:**
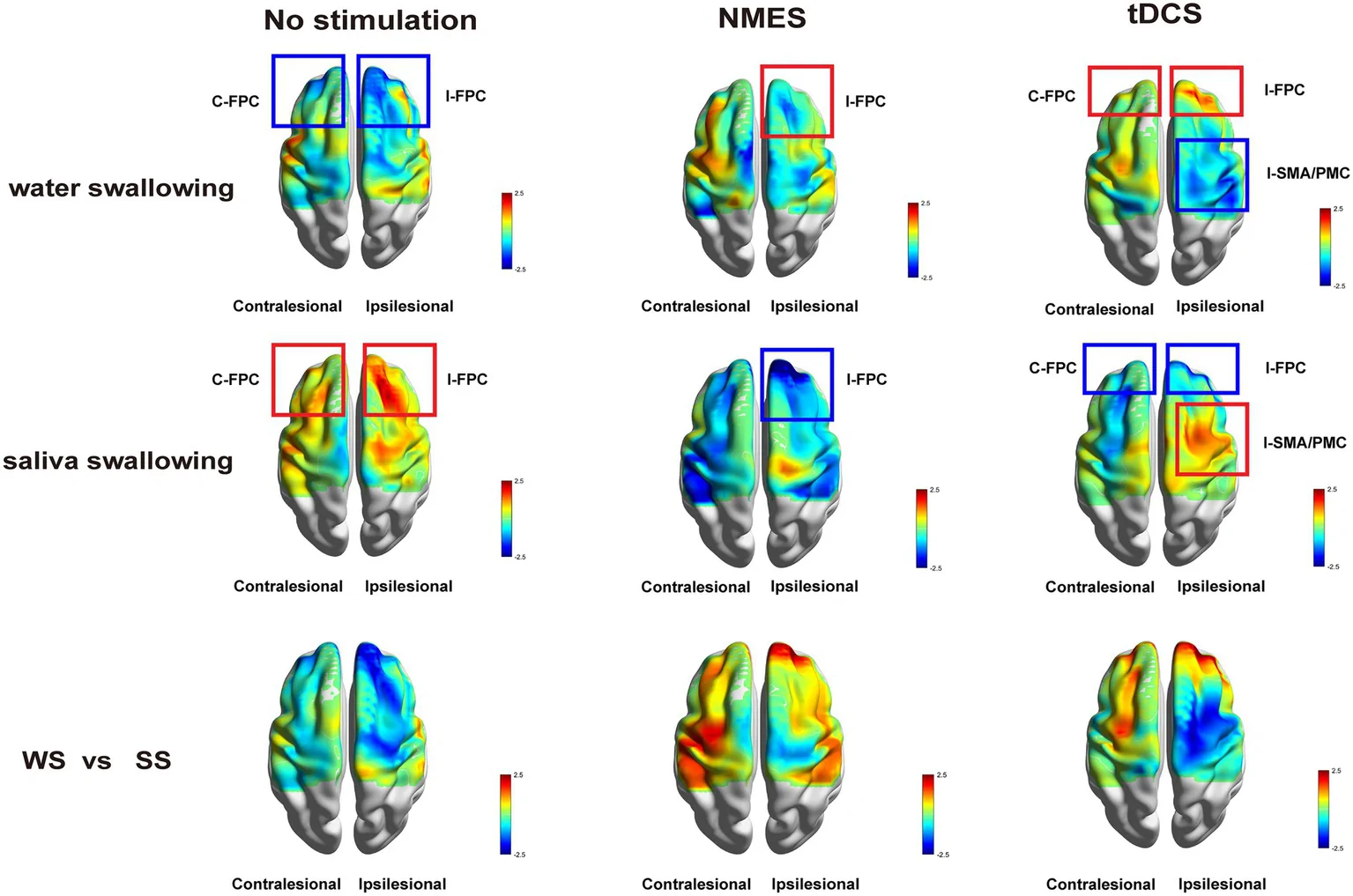
Channel-wise ΔHbO_2_ response patterns for the two swallowing tasks under each stimulation condition. Red boxes highlight cortical regions showing relatively greater task-evoked ΔHbO_2_ responses, whereas blue boxes highlight regions showing relatively lower responses. No stimulation: WS < SS (bilateral FPC); NMES: WS > SS (I-FPC); tDCS: WS > SS (bilateral FPC) and WS < SS (I-SMA/PMC). Highlighted regions correspond to exploratory paired *t*-test comparisons that remained significant after FDR correction within the corresponding comparison family (q < 0.05).

**Figure 3:**
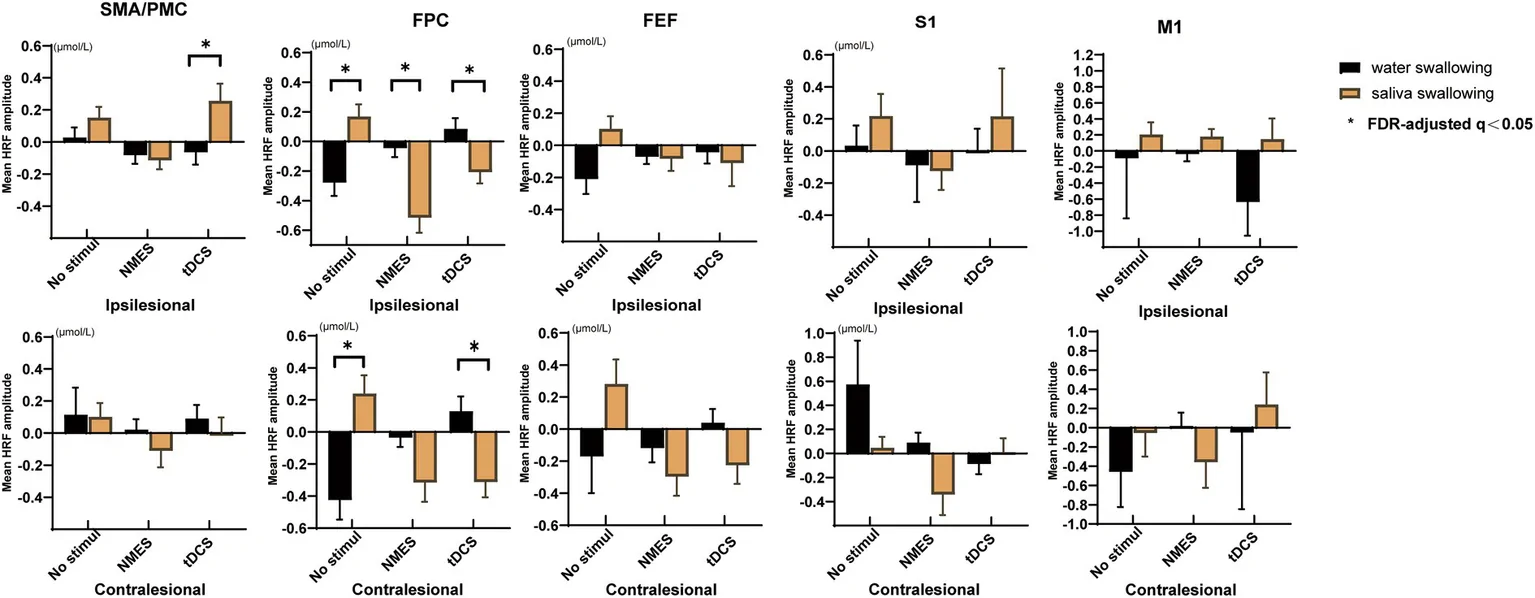
Region-wise ΔHbO₂ differences between swallowing tasks within each stimulation condition based on exploratory paired *t*-tests with FDR correction. In the second set of exploratory analyses, paired *t*-tests were used to compare different intervention conditions within the same swallowing task. Notably, during water swallowing, tDCS was associated with greater bilateral FPC responses than no stimulation (I-FPC: *t* = 3.180, *q* = 0.020; C-FPC: *t* = 4.086, *q* = 0.002). During the saliva swallowing task, tDCS was associated with a greater ΔHbO₂ response in the ipsilesional SMA/PMC than NMES (*t* = 3.430, *q* = 0.014). Compared with no stimulation, NMES was associated with lower responses in the ipsilesional SMA/PMC (*t* = −3.094, *q* = 0.014), bilateral FPC (I-FPC: *t* = −4.741, *q* < 0.001; C-FPC: *t* = −3.967, *q* = 0.002), and contralesional FEF (C-FEF: *t* = −3.137, *q* = 0.020). Similarly, compared with no stimulation, tDCS was associated with lower responses in the bilateral FPC (I-FPC: *t* = −4.013, *q* = 0.001; C-FPC: *t* = −4.026, *q* = 0.001) and contralesional FEF (C-FEF: *t* = −3.139, *q* = 0.026). These paired comparisons were exploratory and remained significant after FDR correction within the corresponding comparison families (Figures 4, 5; Supplementary Table S1).

**Figure 4:**
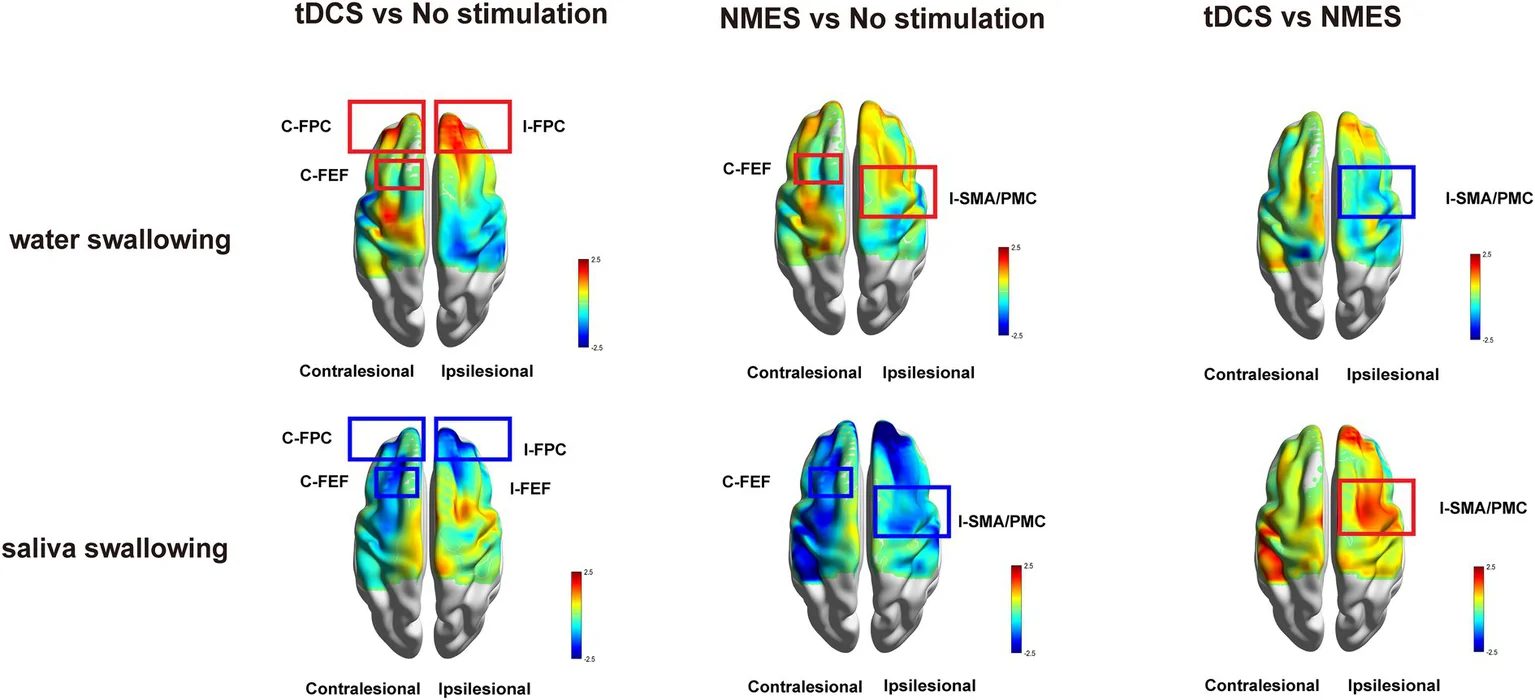
Channel-wise ΔHbO₂ response patterns between stimulation conditions within each swallowing task. Red boxes highlight cortical regions showing relatively greater stimulation-associated ΔHbO₂ responses, whereas blue boxes highlight regions showing relatively lower responses. Water swallowing: tDCS > no stimulation (bilateral FPC). Saliva swallowing: tDCS > NMES (I-SMA/PMC); tDCS < no stimulation (bilateral FPC, C-FEF); NMES < no stimulation (bilateral FPC, C-FEF, I-SMA/PMC). Highlighted regions indicate exploratory paired comparisons that remained significant after FDR correction within the corresponding comparison family (q < 0.05).

**Figure 5:**
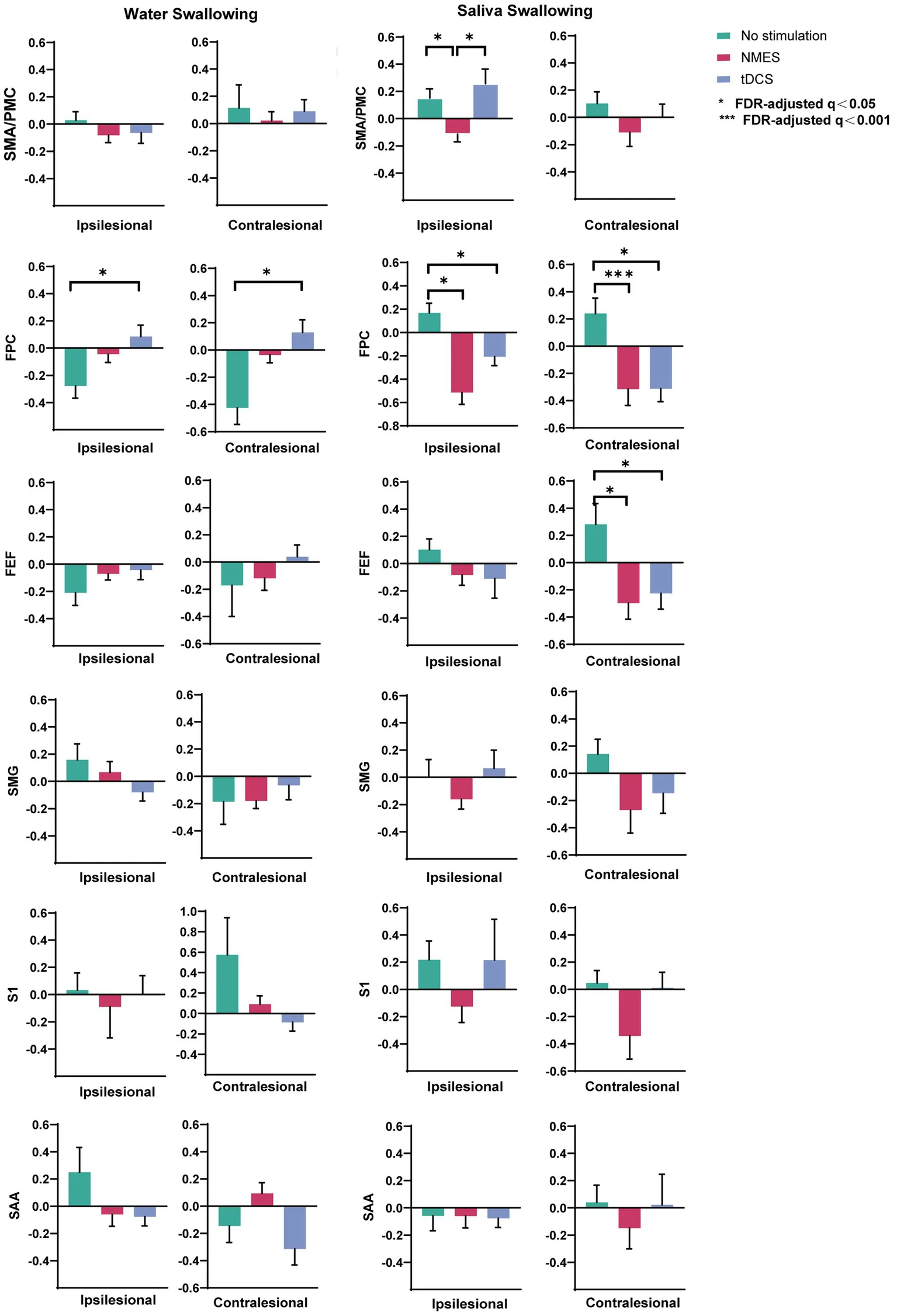
Region-wise ΔHbO_2_ differences between stimulation conditions within each swallowing task based on exploratory paired *t*-tests with FDR correction.

Statistical analyses were carried out using SPSS 27.0 software (IBM SPSS Statistics 27, ©IBM, Armonk, NY, United States). The normality of data distribution and the homogeneity of variance were assessed using the Shapiro–Wilk test, respectively. The primary statistical analysis comprised a 3 (Stimulation: tDCS, NMES, no stimulation) × 2 (Task: water swallowing, saliva swallowing) two-way repeated-measures analysis of variance (RM-ANOVA) conducted for each of the 16 regions of interest (ROIs). The 56 fNIRS channels were grouped into 16 bilateral ROIs based on a predefined anatomical parcellation scheme, and the mean ΔHbO₂ value across channels within each ROI was used as the dependent variable. Mauchly’s test of sphericity was applied, and the Greenhouse–Geisser correction was used when the sphericity assumption was violated. Partial eta squared (η^2^_p_) was reported as a measure of effect size (small: 0.01, medium: 0.06, large: 0.14). Post-hoc pairwise comparisons with Bonferroni correction were performed when a significant main effect or interaction was observed. To further characterize the condition-specific effects identified by the primary RM-ANOVA, exploratory paired-sample *t*-tests were conducted. For the primary ROI-wise RM-ANOVAs, BH-FDR correction was applied separately across the 16 ROIs for each of the three effect families: the Stimulation main effect, Task main effect, and Stimulation × Task interaction. Specifically, paired *t*-tests examined (1) differences between the two swallowing tasks within each intervention condition and (2) differences between intervention conditions within each swallowing task. False discovery rate (FDR) correction was applied separately within each family of comparisons. Throughout the Results and Figures 2–5, FDR-adjusted *p*-values from these exploratory paired comparisons are reported as q-values, and statistical significance was defined as q < 0.05. Raw *p*-values for these exploratory comparisons are not reported unless explicitly stated otherwise. Complete results of all exploratory paired comparisons, including raw *p*-values and FDR-adjusted q-values, are provided in Supplementary Table S1. For clinical outcome measures (KWDT and FOIS), which are ordinal scales, non-parametric analyses were employed. Exact Wilcoxon signed-rank tests were used to compare pre- and post-stimulation scores within each condition. Effect sizes were calculated as r = |Z|/√N. Bootstrap 95% confidence intervals were calculated for the signed post-minus-pre difference rather than for the effect size r. The Friedman test was used to compare changes across the three conditions, followed by pairwise Wilcoxon signed-rank tests with Bonferroni correction. A two-sided significance level of *p* < 0.05 was used for uncorrected analyses. For analyses subjected to FDR correction, statistical significance was defined as q < 0.05. Bonferroni-adjusted post-hoc comparisons were considered significant at p_adj < 0.05.

## 3 Results

Of the 15 enrolled participants, four were excluded during fNIRS preprocessing because excessive motion artifacts prevented reliable estimation of task-related hemodynamic responses. The remaining 11 participants were included in all subsequent analyses.

### 3.1 Primary effects of stimulation and swallowing task on cortical hemodynamic responses

The primary analysis comprised a 3 (Stimulation: tDCS, NMES, no stimulation) × 2 (Task: water swallowing, saliva swallowing) repeated-measures analysis of variance (RM-ANOVA) conducted for each of the 16 regions of interest (ROIs). Only two effects survived correction, both involving the Condition × Task interaction in the FPC. In the ipsilesional FPC, the interaction was significant, *F*(2, 20) = 8.44, raw *p* = 0.002, q = 0.031, partial η^2^ = 0.458. During saliva swallowing, no stimulation produced a higher response than NMES (mean difference = 0.683, Bonferroni-adjusted *p* = 0.043) and tDCS (mean difference = 0.376, adjusted *p* = 0.008). Within tDCS, water swallowing produced a higher response than saliva swallowing (mean difference = 0.292, adjusted *p* = 0.020).

The contralesional FPC showed a comparable interaction, *F*(2, 20) = 7.42, raw *p* = 0.004, q = 0.031, partial η^2^ = 0.426. During water swallowing, tDCS produced a higher response than no stimulation (mean difference = 0.555, adjusted *p* = 0.006). During saliva swallowing, no stimulation produced a higher response than tDCS (mean difference = 0.553, adjusted *p* = 0.034). Within tDCS, water swallowing produced a higher response than saliva swallowing (mean difference = 0.443, adjusted *p* = 0.008).

**Figure 6:**
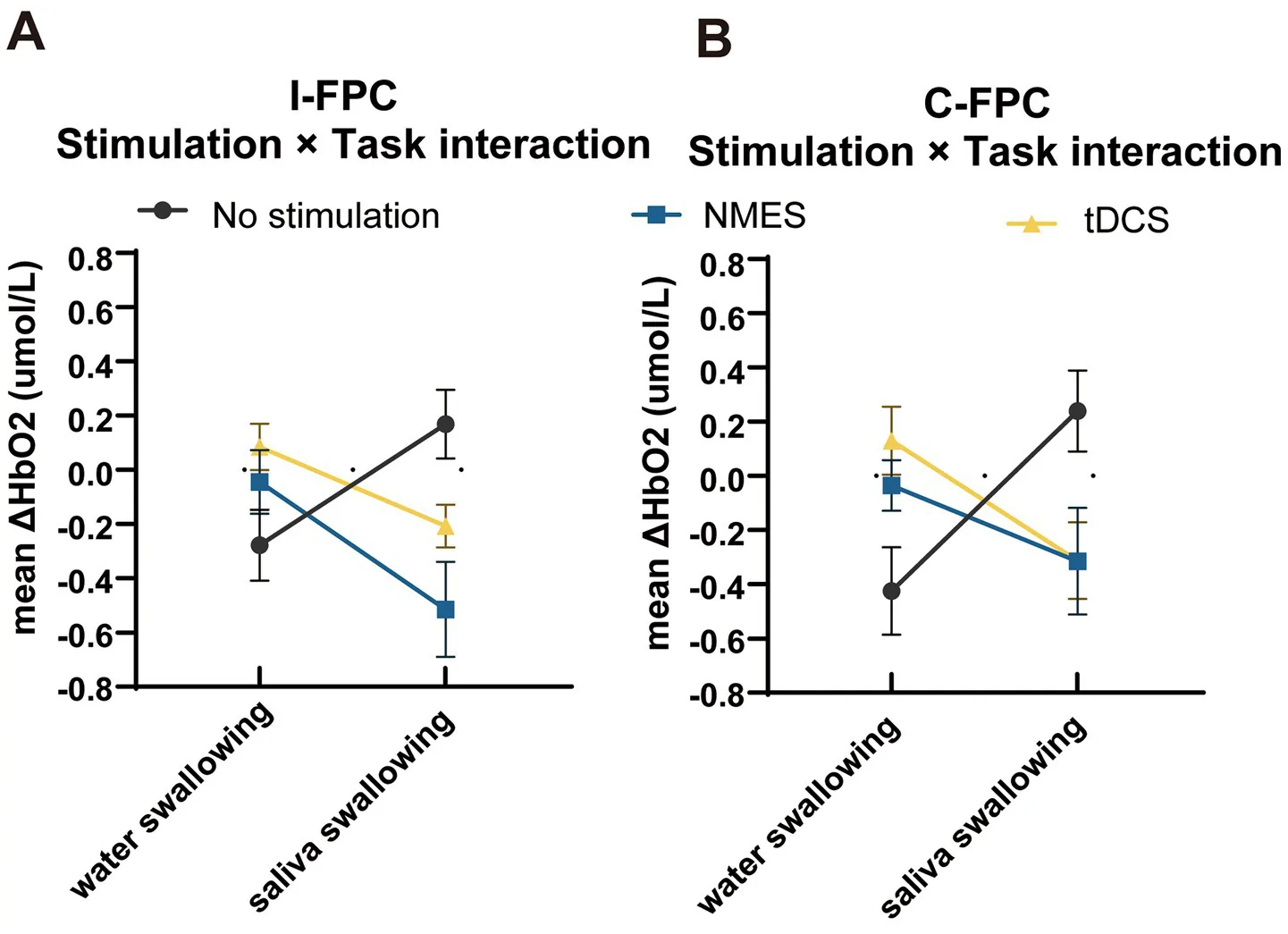
Primary RM-ANOVA effects on cortical hemodynamic responses. Bilateral frontopolar cortex (FPC) Stimulation × Task interactions that survived BH-FDR correction across 16 ROIs. **(A)** Ipsilesional FPC: *F*(2, 20) = 8.44, raw *p* = 0.002, *q* = 0.031. **(B)** Contralesional FPC: *F*(2, 20) = 7.42, raw *p* = 0.004, *q* = 0.031. *I* = ipsilesional; *C* = contralesional after lesion-side mirroring; *WS* = water swallowing; *SS* = saliva swallowing. Bonferroni-adjusted simple effects: **(A)** *SS*, *NS* > NMES (p_adj = 0.043), NS > tDCS (p_adj = 0.008); under tDCS, WS > SS (p_adj = 0.020). **(B)** WS, tDCS > NS (p_adj = 0.006); SS, NS > tDCS (p_adj = 0.034); under tDCS, WS > SS (p_adj = 0.008).

The contralesional FEF Condition effect, *F*(2, 20) = 4.08, raw *p* = 0.033, q = 0.524, and ipsilesional M1 Task effect, *F*(1, 10) = 5.41, raw *p* = 0.042, q = 0.464, did not survive ROI-level FDR and were therefore treated as nominal exploratory findings rather than primary results. No other ROI-level effect survived correction (Figure 6; Table 2).

**Table 2:** Summary of significant repeated-measures ANOVA results for region-wise ΔHbO₂ levels across stimulation conditions and swallowing tasks.

ROI	Significant effect	*F*(df)	Raw p	BH-FDR q	η^2^_p_	Effect Size	Post-hoc (Bonferroni)
I-FPC	Stimulation × Task interaction	*F*(2, 20) = 8.44	0.002	0.031	0.458	Large	SS: No Stimulation > tDCS, p_adj = 0.008;
							No Stimulation > NMES, p_adj = 0.043;
							tDCS: WS > SS, p_adj = 0.02
C-FPC	Stimulation × Task interaction	*F*(2, 20) = 7.42	0.004	0.031	0.426	Large	WS: No Stimulation < tDCS, p_adj = 0.006;
							SS: No Stimulation > tDCS, p_adj = 0.034;
							tDCS: WS > SS, p_adj = 0.008

RM-ANOVA = repeated-measures analysis of variance; I-FPC = right frontopolar cortex; C-FPC = left frontopolar cortex (after lesion-side mirroring); *F*(df) = *F*-statistic (degrees of freedom). Raw *p* values are from the ROI-wise RM-ANOVA. q values represent BH-FDR-adjusted *p* values across the 16 ROIs within the corresponding effect family. η^2^_p_ = partial eta squared. Simple-effect p_adj values were adjusted using the Bonferroni method.

To further characterize the condition-specific effects identified by the primary RM-ANOVA, exploratory paired *t*-tests with false discovery rate (FDR) correction were conducted as post-hoc analyses. These analyses examined (1) differences between the two swallowing tasks within each intervention condition, and (2) differences between intervention conditions within each swallowing task. All paired *t*-test results should be interpreted as exploratory given the preliminary nature of this pilot study and the limited sample size. Complete results for all exploratory paired comparisons, including both significant and non-significant findings, raw *p*-values, and BH-FDR-adjusted q-values, are provided in Supplementary Table S1.

In the first set of exploratory analyses, paired *t*-tests were conducted to compare the two swallowing tasks within each intervention condition. Under the no-stimulation condition, bilateral FPC ΔHbO₂ responses were lower during water swallowing than during saliva swallowing (I-FPC: *t* = −3.44, q = 0.018; C-FPC: *t* = −3.108, q = 0.024). During the NMES condition, water swallowing was associated with a greater ΔHbO₂ response in the ipsilesional FPC than saliva swallowing (*t* = 4.152, q = 0.002). During tDCS, ipsilesional SMA/PMC responses were greater during saliva than water swallowing (*t* = −3.325, q = 0.005), whereas bilateral FPC responses were greater during water than saliva swallowing (I-FPC: *t* = 3.859, q = 0.002; C-FPC: *t* = 4.119, q = 0.002). All reported q-values were FDR-adjusted within the corresponding family of exploratory comparisons (Figures 2, 3).

In the second set of exploratory analyses, paired *t*-tests were used to compare different intervention conditions within the same swallowing task. Notably, during water swallowing, tDCS was associated with greater bilateral FPC responses than no stimulation (I-FPC: t = 3.180, q = 0.020; C-FPC: t = 4.086, q = 0.002). During the saliva swallowing task, tDCS was associated with a greater ΔHbO₂ response in the ipsilesional SMA/PMC than NMES (t = 3.430, q = 0.014). Compared with no stimulation, NMES was associated with lower responses in the ipsilesional SMA/PMC (t = −3.094, q = 0.014), bilateral FPC (I-FPC: t = −4.741, q < 0.001; C-FPC: t = −3.967, q = 0.002), and contralesional FEF (C-FEF: t = −3.137, q = 0.020). Similarly, compared with no stimulation, tDCS was associated with lower responses in the bilateral FPC (I-FPC: t = −4.013, q = 0.001; C-FPC: t = −4.026, q = 0.001) and contralesional FEF (C-FEF: t = −3.139, q = 0.026). These paired comparisons were exploratory and remained significant after FDR correction within the corresponding comparison families (Figures 4, 5; Supplementary Table S1).

### 3.2 Hemispheric lateralization

Laterality indices were calculated at the participant level for eight homologous ROI pairs and analyzed using separate 3 (Condition) × 2 (Task) RM-ANOVAs. After BH-FDR correction across the eight ROI pairs within each effect family, no Condition, Task, or Condition × Task effect remained significant (all q > 0.05; Table 3). The smallest unadjusted *p* value was observed for the DLPFC Condition effect, *F*(2, 20) = 4.06, *p* = 0.033, but this effect did not survive correction (q = 0.266). Accordingly, the present data do not provide FDR-supported evidence for task- or stimulation-dependent hemispheric lateralization.

**Table 3:** Laterality-index analysis across stimulation conditions and swallowing tasks.

Stimulation–task	SMG	SMA/PMC	M1	SAA	S1	DLPFC	FEF	FPC
NS–WS	0.286 ± 0.674	0.301 ± 0.598	0.201 ± 0.710	0.434 ± 0.600	−0.073 ± 0.780	0.366 ± 0.776	−0.011 ± 0.727	0.141 ± 0.703
NS–SS	0.024 ± 0.877	0.128 ± 0.626	0.180 ± 0.561	0.011 ± 0.766	0.383 ± 0.810	0.184 ± 0.666	−0.103 ± 0.643	−0.244 ± 0.645
NMES–WS	0.282 ± 0.804	−0.221 ± 0.848	−0.064 ± 0.823	−0.297 ± 0.586	−0.266 ± 0.741	−0.334 ± 0.644	−0.041 ± 0.632	−0.164 ± 0.691
NMES–SS	−0.151 ± 0.767	−0.127 ± 0.415	0.392 ± 0.730	−0.017 ± 0.770	0.028 ± 0.755	−0.184 ± 0.766	0.379 ± 0.438	−0.309 ± 0.650
tDCS–WS	0.059 ± 0.603	−0.347 ± 0.782	0.079 ± 0.936	0.251 ± 0.729	0.235 ± 0.854	0.336 ± 0.812	−0.274 ± 0.771	0.019 ± 0.896
tDCS–SS	0.086 ± 0.681	0.298 ± 0.577	−0.057 ± 0.607	0.383 ± 0.562	0.137 ± 0.805	−0.043 ± 0.801	0.158 ± 0.720	−0.033 ± 0.686
Condition effect	*F*(2, 20) = 0.11; *p* = 0.899; q = 0.899	*F*(2, 20) = 2.05; *p* = 0.155; q = 0.414	*F*(2, 20) = 0.33; *p* = 0.722; q = 0.825	*F*(2, 20) = 2.45; *p* = 0.112; q = 0.414	*F*(2,20) = 1.68; *p* = 0.212; q = 0.424	*F*(2, 20) = 4.06; *p* = 0.033; q = 0.266	*F*(1.3, 13.3) = 1.11; *p* = 0.332; q = 0.532	*F*(2, 20) = 0.62; *p* = 0.549; q = 0.732
Task effect	*F*(1, 10) = 1.34; *p* = 0.274; q = 0.438	*F*(1, 10) = 1.36; *p* = 0.271; q = 0.438	*F*(1, 10) = 0.51; *p* = 0.490; q = 0.560	*F*(1, 10) = 0.00; *p* = 0.984; q = 0.984	*F*(1, 10) = 1.58; *p* = 0.237; q = 0.438	*F*(1, 10) = 0.75; *p* = 0.408; q = 0.544	*F*(1, 10) = 1.54; *p* = 0.243; q = 0.438	*F*(1, 10) = 1.69; *p* = 0.223; q = 0.438
Condition × Task interaction	*F*(2, 20) = 0.56; *p* = 0.580; q = 0.680	*F*(2, 20) = 2.30; *p* = 0.126; q = 0.680	*F*(2, 20) = 1.12; *p* = 0.347; q = 0.680	*F*(2, 20) = 1.61; *p* = 0.225; q = 0.680	*F*(2, 20) = 0.53; *p* = 0.595; q = 0.680	*F*(2, 20) = 0.54; *p* = 0.593; q = 0.680	*F*(1.1, 11.4) = 1.27; *p* = 0.291; q = 0.680	*F*(2, 20) = 0.29; *p* = 0.754; q = 0.754

Cell values are mean LI ± SD. Positive LI denotes ipsilesional predominance and negative LI denotes contralesional predominance. *P* values are raw RM-ANOVA *p* values; q values are BH-FDR-adjusted values across the eight homologous ROI pairs within each effect family. No Condition, Task, or interaction effect survived FDR (all q > 0.05). The nominal DLPFC Condition effect (*p* = 0.033) did not survive correction (q = 0.266).

### 3.3 Clinical outcome measures

Clinical swallowing outcomes were assessed before and after each stimulation condition using exact Wilcoxon signed-rank tests. For KWDT, no change was observed under the no-stimulation condition (0/11 patients improved, *p* = 1.000, r = 0.000). Four of 11 patients improved following NMES and four following tDCS; neither pre–post comparison reached statistical significance (NMES: Z = -1.643, *p* = 0.125, r = 0.495; tDCS: Z = -1.643, *p* = 0.125, r = 0.495). For both stimulation conditions, the median change was 0, with a bootstrap 95% CI of [−1, 0] for the signed post-minus-pre difference. The Friedman test did not show a significant overall difference in KWDT change across conditions, χ^2^(2) = 5.33, *p* = 0.069, Kendall’s W = 0.242, and none of the Bonferroni-corrected pairwise comparisons reached significance.

For FOIS, no change was observed under the no-stimulation condition (0/11 patients improved, *p* = 1.000, r = 0.000). Four of 11 patients improved following NMES (Z = -1.643, p = 0.125, r = 0.495), and five of 11 improved following tDCS (Z = -1.888, *p* = 0.063, r = 0.569); neither within-condition comparison reached statistical significance. The median change was 0 for both NMES and tDCS, with bootstrap 95% CIs of [0, 1]. Although the Friedman test indicated an overall difference in FOIS change across conditions, χ^2^(2) = 8.40, *p* = 0.015, Kendall’s W = 0.382, none of the Bonferroni-corrected pairwise comparisons reached statistical significance (all adjusted *p* > 0.05) (Supplementary Table S2).

## 4 Discussion

Our preliminary findings suggest that cortical hemodynamic responses differed between swallowing tasks and varied across stimulation conditions. The strongest statistical evidence was provided by the bilateral FPC Stimulation × Task interactions, whereas the additional ROI-wise paired comparisons should be regarded as exploratory and hypothesis-generating. These findings raise questions regarding possible task-dependent responses to neuromodulation but do not establish distinct physiological mechanisms or support selection of a specific intervention for an individual swallowing deficit.

### 4.1 Task-dependent cortical processing of swallowing

A foundational finding of our study is the differential cortical hemodynamic responses between saliva and water swallowing in the non-stimulated baseline condition. Saliva swallowing elicited significantly greater activation in the bilateral FPC compared to water swallowing. This finding is consistent with the view that saliva swallowing is a more internally-driven, volitional act requiring significant involvement of higher-order cognitive processes, including intention, planning, and initiation (Jones et al., 2020). From a neuroscience perspective, swallowing water is considered a “sensory-induced” action, relying on the physical stimuli (volume, pressure, temperature) generated by the liquid on the oral-pharyngeal mucosa to trigger a cascade of swallowing reflexes. Swallowing saliva, on the other hand, is a typical example of an “autonomously initiated” action, highly dependent on the generation of endogenous motor intentions (Tsuchiya et al., 2023). This finding suggests that in the absence of explicit external sensory input, swallowing saliva relies more on advanced motor planning and self-initiation mechanisms (Cheng et al., 2022). The FPC, as part of the prefrontal cortex, is integral to these executive functions. In contrast, water swallowing provides additional oropharyngeal sensory input from the liquid bolus and may therefore involve different sensory and motor demands from cued saliva swallowing. While it still requires cortical oversight, the reduced demand on volitional planning may explain the lower FPC activation observed. This task-dependent distinction underscores that not all swallowing acts are processed identically by the brain, a critical consideration when designing and evaluating rehabilitation interventions for post-stroke dysphagia, a condition known to affect over half of acute stroke patients (Dziewas et al., 2021).

### 4.2 Divergent task-dependent cortical responses associated with NMES and tDCS

Exploratory paired comparisons suggested different task-dependent response patterns under NMES and tDCS.

For NMES, exploratory comparisons showed a greater ipsilesional FPC response during water swallowing than during saliva swallowing. Given that NMES provides peripheral sensory input, this finding may reflect differences in how such input interacts with cortical processing across swallowing tasks (Jones et al., 2020). However, afferent sensory activity and sensorimotor integration were not directly measured in this study (Chen et al., 2016; Alamer et al., 2020). It therefore remains unclear whether the observed response reflects enhanced sensory–motor integration, reduced volitional motor demand, or other processes. Our findings simply suggest that the cortical response associated with NMES may vary with the sensory and motor demands of the swallowing task.

In contrast, tDCS was associated with task-dependent hemodynamic changes involving the bilateral FPC, while exploratory comparisons also identified differences in the ipsilesional SMA/PMC. tDCS is generally considered to influence cortical excitability through changes in neuronal membrane potentials (Adeyemo et al., 2012), and previous studies have reported effects on swallowing-related cortical activity and functional networks (Chen et al., 2016; Alamer et al., 2020; Adeyemo et al., 2012; Wang et al., 2023). In the present study, the FPC response differed according to swallowing task, with greater responses during water than saliva swallowing under tDCS, while the exploratory SMA/PMC findings suggested additional task-related differences in motor-related regions. Considering the roles of the FPC and SMA/PMC in higher-order control and motor planning, these findings may reflect differences in cortical processing under different task demands (Yang et al., 2012; Marchina et al., 2021). Importantly, however, the hemodynamic response was not uniformly increased across tasks or regions. This pattern suggests that the cortical responses associated with tDCS may be task dependent rather than reflecting a generalized facilitation of cortical activity (Polanía et al., 2011). Because fNIRS measures hemodynamic responses rather than neuronal excitability or functional connectivity directly, the underlying physiological mechanisms cannot be determined from the present data.

Overall, NMES and tDCS were associated with different task-dependent patterns of cortical hemodynamic responses. These findings provide a basis for considering how peripheral and central neuromodulation may interact differently with swallowing-related cortical processing, but they do not establish distinct physiological mechanisms for the two interventions. Further sham-controlled studies using methods that directly assess neural activity and network connectivity are needed to clarify the mechanisms underlying these observations.

### 4.3 RM-ANOVA evidence for task-stimulation interaction in bilateral frontopolar cortex

The repeated-measures ANOVA provided the primary statistical evidence for the task-dependent nature of stimulation effects. The most robust finding was the Stimulation × Task interaction in the bilateral frontopolar cortex (FPC), with large effect sizes in both the contralesional FPC (*F*(2, 20) = 7.42, *p* = 0.004, q = 0.031, partial η^2^ = 0.426) and ipsilesional FPC (*F*(2, 20) = 8.44, *p* = 0.002, q = 0.031, partial η^2^ = 0.458). Although these interaction effects were large according to conventional partial η^2^ benchmarks, their magnitude should be interpreted cautiously because effect-size estimates from small exploratory samples are inherently imprecise and may attenuate in larger replication studies. This crossover interaction pattern indicated that the effect of stimulation condition on cortical hemodynamic responses varied according to the swallowing task. During water swallowing, tDCS produced the highest activation in both FPC regions, while during saliva swallowing, the no-stimulation condition yielded the highest activation, with tDCS and NMES producing marked deactivation. The consistency of this crossover pattern across both hemispheres suggests that the bilateral FPC is specifically sensitive to the interaction between external neuromodulatory input and task-specific cognitive demand, rather than responding to either factor in isolation (Zheng et al., 2025; Bradley et al., 2022). This interpretation is consistent with recent neuromodulation studies demonstrating that stimulation effects within the frontoparietal control system are expressed in a task-dependent manner, emerging preferentially during cognitively demanding executive processes rather than reflecting nonspecific increases in cortical hemodynamic responses. These findings identify the bilateral FPC as the principal region showing FDR-supported task-dependent modulation in the present dataset (Cheng et al., 2023).

### 4.4 Laterality analysis

Laterality-index analyses did not identify FDR-supported changes in hemispheric balance across stimulation conditions or swallowing tasks. After correction across the eight homologous ROI pairs, no main effect of Condition, main effect of Task, or Condition × Task interaction remained significant. Although the DLPFC showed a nominal Condition effect, *F*(2, 20) = 4.06, *p* = 0.033, this effect did not survive multiple-comparison correction (q = 0.266) and should therefore not be interpreted as evidence of stimulation-dependent lateralization.

The absence of corrected laterality effects indicates that the acute hemodynamic responses observed under the NMES and tDCS conditions were not accompanied by a consistent redistribution of activity between the ipsilesional and contralesional hemispheres. This finding is compatible with the bilateral and distributed organization of cortical swallowing control, in which task-related responses may vary within individual regions without producing a systematic shift in hemispheric predominance. However, the substantial inter-individual variability in LI values, together with the small sample size and heterogeneous lesion locations, may also have limited the ability to detect reproducible lateralization effects. Larger studies incorporating lesion-stratified analyses, sham stimulation, and repeated treatment sessions are required to determine whether neuromodulation produces reliable changes in hemispheric balance during swallowing after stroke.

### 4.5 Clinical implications and future directions

The clinical findings of the present study should be interpreted cautiously. Although the Friedman test showed an overall difference in FOIS changes across the three conditions, none of the Bonferroni-corrected pairwise comparisons reached statistical significance. No significant pre–post changes were observed in KWDT. Therefore, the observed FOIS changes should be regarded as preliminary directional observations rather than evidence of a clinically meaningful treatment effect after a single stimulation session. Previous studies have reported improvements in swallowing function following repeated NMES or tDCS treatment (Zhu and Gu, 2022; Bengisu et al., 2024). These studies provide useful clinical context, but differences in treatment duration and study design preclude direct comparison with the present single-session findings.

The limited clinical changes observed here may partly reflect the acute nature of the study, in which cortical hemodynamic responses were assessed after a single stimulation session. Whether these acute cortical responses are related to subsequent functional recovery remains unknown. Rather, they show that cortical hemodynamic responses varied according to both stimulation condition and swallowing task, raising the question of whether these acute response patterns have any relevance to longer-term rehabilitation outcomes.

Post-stroke dysphagia is clinically heterogeneous, and patients may differ in the relative contribution of sensory, motor, and volitional deficits to their swallowing impairment (Cohen et al., 2016). In this context, the task-dependent cortical patterns observed in the present study suggest that the interaction between swallowing behavior and neuromodulation may be worth considering in future rehabilitation research. For example, future studies could examine whether patients with different swallowing deficits respond differently to peripheral or central stimulation. However, the present study did not demonstrate differential clinical efficacy between NMES and tDCS, and therefore does not support selecting a particular stimulation strategy for a specific swallowing deficit at this stage. The proposed task–stimulation matching framework should be regarded as a hypothesis for future testing.

Another question is whether peripheral and central stimulation might be used sequentially or in combination. Previous studies have reported promising clinical findings with combined NMES and tDCS approaches (El Nahas et al., 2024; Zhang et al., 2019), providing a basis for further investigation. The regionally differentiated cortical responses observed in the present study raise the possibility that peripheral and central neuromodulation may engage partially distinct components of the swallowing network. If confirmed in future studies, such differences could provide a rationale for examining whether simultaneous or sequential application of these interventions produces broader modulation of swallowing-related networks than either modality alone. However, the present findings do not demonstrate complementary mechanisms or synergistic clinical effects (Dziewas et al., 2021; Bengisu et al., 2024), nor do they establish a mechanistic explanation for the clinical benefits reported in previous combined-intervention studies. Future sham-controlled studies with repeated treatment sessions are needed to determine whether combined or task-specific stimulation produces reproducible neurophysiological changes and, more importantly, whether these changes are accompanied by meaningful improvements in swallowing function.

### 4.6 Limitations and future directions

This exploratory pilot study has several important limitations that should be considered when interpreting the findings. First, the small analyzed sample (*n* = 11), together with a 27% participant exclusion rate, limited statistical precision and increased uncertainty in effect-size estimation. Although four participants were excluded because excessive motion artifacts prevented reliable estimation of task-related hemodynamic responses, no obvious differences in age, sex, lesion location, stroke type, or swallowing severity were observed between the excluded and analyzed participants (Table 1). Nevertheless, given the limited sample size, potential selection bias cannot be completely excluded. In addition, although the Stimulation × Task interactions in the bilateral FPC were associated with large partial η^2^ values (0.458 for the ipsilesional FPC and 0.426 for the contralesional FPC), these estimates were derived from a very small exploratory sample and should therefore be interpreted cautiously. Effect-size estimates from small pilot studies are inherently imprecise and may overestimate the magnitude of the corresponding population effects; thus, the observed effect sizes may attenuate in larger replication samples. The repeated-measures design and multiple-comparison correction improve the robustness of the statistical inference but do not eliminate uncertainty in effect-size estimation. Consequently, the present findings should be regarded as preliminary and hypothesis-generating rather than confirmatory or as stable estimates of the magnitude of task-dependent cortical modulation. Larger, adequately powered studies are required to validate the observed task-dependent cortical hemodynamic response patterns and to obtain more precise estimates of their effect sizes.

Second, the absence of sham-controlled conditions limits the ability to distinguish stimulation-specific effects from nonspecific cognitive or sensory influences. Recent conceptual frameworks emphasize that neuromodulatory effects are highly state-dependent and cannot be reliably distinguished from nonspecific cognitive or sensory influences without appropriate control conditions (Wang et al., 2023). Accordingly, all stimulation-related findings should be interpreted as acute hemodynamic associations rather than definitive evidence of stimulation-specific neural modulation.

Third, lesion location and stroke subtype were heterogeneous across participants, encompassing cortical and subcortical lesions in both hemispheres as well as ischemic and hemorrhagic stroke. Such heterogeneity is likely to influence the organization of the swallowing network and responsiveness to neuromodulation, may also contribute to the variability observed in FPC activation patterns and hemispheric lateralization indices. Although the within-subject crossover design reduced inter-individual variability, future studies should stratify participants according to lesion characteristics or incorporate lesion mapping to better define stimulation-response relationships.

Fourth, the bilateral tDCS montage used in the present study differs from the unilateral stimulation protocols more commonly employed in post-stroke dysphagia rehabilitation. Previous studies have investigated bilateral stimulation of swallowing-related motor cortices, providing general support for the feasibility of bilateral cortical targeting after stroke (Ahn et al., 2017; Lindenberg et al., 2010). However, the specific C3/C4 anodal–FC6 return configuration used here was a study-specific experimental design and differed from previously reported bilateral stimulation protocols. Moreover, recent evidence indicates that the relative benefit of bilateral versus unilateral or sham stimulation remains uncertain, and the optimal electrode configuration has not been established. Because computational electric-field modeling was not performed, the precise spatial distribution of the induced electric field cannot be determined from the present data. Future studies combining individualized electric-field modeling with sham-controlled comparisons are needed to optimize bilateral stimulation targeting and determine its clinical relevance.

Fifth, this study evaluated only the immediate cortical and clinical effects of a single stimulation session. Although a trend toward improved FOIS scores was observed following tDCS, no intervention produced statistically significant clinical improvement. This discrepancy between measurable cortical hemodynamic modulation and limited functional change is consistent with current models of neuromodulation, in which alterations in cortical network activity are expected to precede observable behavioral recovery. Therefore, future longitudinal studies incorporating repeated stimulation sessions are required to determine whether the task-dependent cortical responses identified in the present study predict long-term improvements in swallowing function.

Sixth, although a washout interval of at least 24 h was implemented between stimulation sessions, the possibility of residual carry-over effects, particularly following tDCS, cannot be completely excluded because empirical evidence regarding the optimal washout duration remains limited. Future crossover studies should consider longer washout intervals or parallel-group randomized designs to minimize potential carry-over effects. In addition, the order of the swallowing tasks was fixed rather than randomized, with water swallowing performed before saliva swallowing in each session. Although a rest interval was introduced between tasks, residual effects including practice, fatigue, anticipatory processing, or carry-over activation cannot be completely excluded. Because the principal finding of this study was a task-dependent cortical interaction, future studies should counterbalance task order to confirm that the observed differences reflect swallowing behavior rather than sequence effects.

Finally, fNIRS measures only cortical hemodynamic activity and cannot directly assess the subcortical and brainstem structures that are critically involved in swallowing control. Multimodal approaches integrating fNIRS with EEG or MRI may provide complementary spatial and temporal information on stimulation-induced cortical dynamics (Cohen et al., 2016).

**Figure 7:**
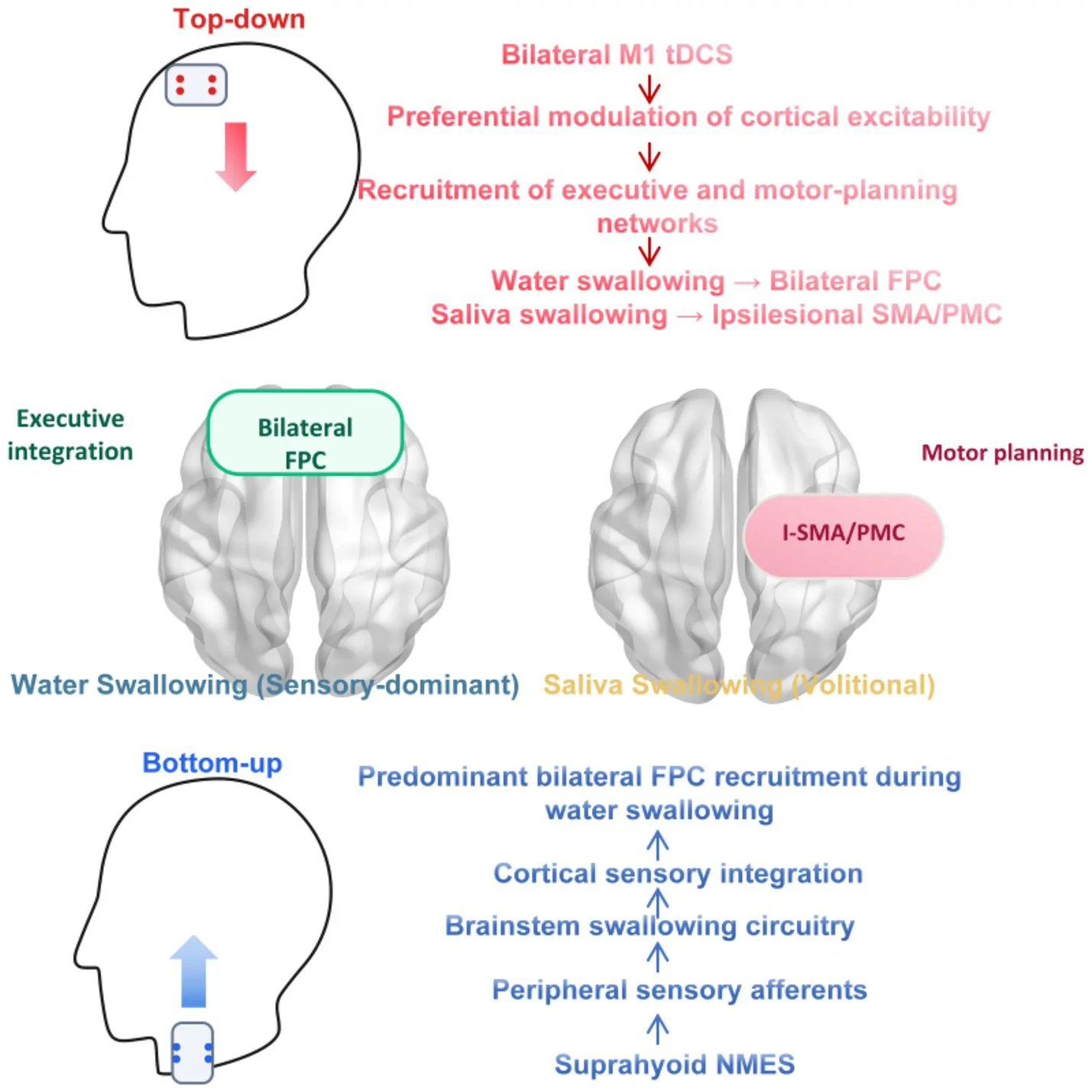
Summarizes the principal findings of this exploratory pilot study. Peripheral NMES is proposed to modulate swallowing-related cortical activity primarily through bottom-up sensory afferent pathways, whereas bilateral M1 tDCS preferentially influences executive and motor-planning networks through top-down modulation of cortical excitability. Water swallowing was associated predominantly with bilateral frontopolar cortex (FPC) recruitment, whereas saliva swallowing preferentially engaged the ipsilesional supplementary motor area/premotor cortex (SMA/PMC). These observations suggest that cortical responses to neuromodulation depend on both stimulation modality and swallowing task, supporting a task-dependent organization of the cortical swallowing network.

Overall, the present pilot study provides preliminary evidence that cortical responses to neuromodulation depend on the interaction between stimulation modality and swallowing behavior. Future studies should use adequately powered, sham-controlled longitudinal designs with repeated stimulation sessions and clinically meaningful swallowing outcomes. Lesion-stratified analyses, counterbalanced task order, short-separation fNIRS measurements, and multimodal neuroimaging may further clarify the reproducibility and physiological basis of the observed task-dependent cortical responses (Figure 7).

## 5 Conclusion

This exploratory pilot study provides preliminary evidence that peripheral (NMES) and central (tDCS) neuromodulation elicit distinct acute cortical hemodynamic responses during swallowing after stroke, with their effects varying according to both stimulation modality and swallowing task. Rather than uniformly altering cortical hemodynamic responses, NMES and tDCS preferentially engaged different components of the swallowing network, supporting a functionally differentiated and task-dependent organization of cortical swallowing control. Importantly, these findings reflect acute neurophysiological modulation rather than evidence of long-term neuroplasticity or therapeutic superiority. Although limited by the small sample size, the absence of sham stimulation, and the single-session design, this study provides preliminary mechanistic insight into how peripheral and central neuromodulation may engage potentially partially distinct cortical mechanisms during swallowing. Future adequately powered, sham-controlled longitudinal studies incorporating repeated stimulation protocols, lesion-stratified analyses, and multimodal neuroimaging are warranted to determine whether these acute cortical hemodynamic responses translate into sustained functional recovery and to further evaluate the proposed task–stimulation matching hypothesis.

## Data Availability

The raw data supporting the conclusions of this article will be made available by the authors, without undue reservation.
