# Supplementary material for: Task-dependent cortical responses to peripheral and central neuromodulation during water and saliva swallowing after stroke: an exploratory fNIRS pilot study
Source: Front Hum Neurosci. 2026 Aug 24;20:1907700. doi: 10.3389/fnhum.2026.1907700 (PMC13547267; doi:10.3389/fnhum.2026.1907700)
Supplement: Supplementary file 1 [file Table_1.docx]

**Supplementary Table S1. Exploratory Paired Comparisons**

| **Family ID** | **Comparison type** | **Condition/task** | **Contrast** | **ROI** | **t statistic** | **Raw p** | **BH-FDR q** | **Significant after correction** |
| --- | --- | --- | --- | --- | --- | --- | --- | --- |
| F1 | Task comparison within condition | tDCS | WS vs SS | I-SMG | -1.05 | 0.298 | 0.580 | No |
| F1 | Task comparison within condition | tDCS | WS vs SS | C-SMG | 0.42 | 0.677 | 0.677 | No |
| **F1** | **Task comparison within condition** | **tDCS** | **WS vs SS** | **I-SMA/PMC** | **-3.33** | **0.001** | **0.005** | **Yes** |
| F1 | Task comparison within condition | tDCS | WS vs SS | C-SMA/PMC | 0.56 | 0.578 | 0.661 | No |
| F1 | Task comparison within condition | tDCS | WS vs SS | I-M1 | -2.06 | 0.067 | 0.268 | No |
| F1 | Task comparison within condition | tDCS | WS vs SS | C-M1 | -0.67 | 0.52 | 0.661 | No |
| F1 | Task comparison within condition | tDCS | WS vs SS | I-SAA | -1.37 | 0.181 | 0.418 | No |
| F1 | Task comparison within condition | tDCS | WS vs SS | C-SAA | -1.36 | 0.183 | 0.418 | No |
| F1 | Task comparison within condition | tDCS | WS vs SS | I-S1 | -0.625 | 0.537 | 0.661 | No |
| F1 | Task comparison within condition | tDCS | WS vs SS | C-S1 | -0.739 | 0.466 | 0.661 | No |
| F1 | Task comparison within condition | tDCS | WS vs SS | I-DLPFC | 0.604 | 0.559 | 0.661 | No |
| F1 | Task comparison within condition | tDCS | WS vs SS | C-DLPFC | -1.032 | 0.326 | 0.580 | No |
| F1 | Task comparison within condition | tDCS | WS vs SS | I-FEF | 0.443 | 0.663 | 0.677 | No |
| F1 | Task comparison within condition | tDCS | WS vs SS | C-FEF | 1.674 | 0.109 | 0.349 | No |
| **F1** | **Task comparison within condition** | **tDCS** | **WS vs SS** | **I-FPC** | **3.859** | **0.0003** | **0.002** | **Yes** |
| **F1** | **Task comparison within condition** | **tDCS** | **WS vs SS** | **C-FPC** | **4.119** | **0.0001** | **0.002** | **Yes** |
| F2 | Task comparison within condition | NMES | WS vs SS | I-SMG | 1.937 | 0.059 | 0.276 | No |
| F2 | Task comparison within condition | NMES | WS vs SS | C-SMG | 0.534 | 0.596 | 0.794 | No |
| F2 | Task comparison within condition | NMES | WS vs SS | I-SMA/PMC | 0.398 | 0.691 | 0.8 | No |
| F2 | Task comparison within condition | NMES | WS vs SS | C-SMA/PMC | 1.22 | 0.225 | 0.4 | No |
| F2 | Task comparison within condition | NMES | WS vs SS | I-M1 | -1.527 | 0.158 | 0.4 | No |
| F2 | Task comparison within condition | NMES | WS vs SS | C-M1 | 1.368 | 0.201 | 0.4 | No |
| F2 | Task comparison within condition | NMES | WS vs SS | I-SAA | 0.388 | 0.7 | 0.8 | No |
| F2 | Task comparison within condition | NMES | WS vs SS | C-SAA | 1.236 | 0.225 | 0.4 | No |
| F2 | Task comparison within condition | NMES | WS vs SS | I-S1 | 0.133 | 0.895 | 0.905 | No |
| F2 | Task comparison within condition | NMES | WS vs SS | C-S1 | 2.060 | 0.048 | 0.276 | No |
| F2 | Task comparison within condition | NMES | WS vs SS | I-DLPFC | 0.627 | 0.545 | 0.793 | No |
| F2 | Task comparison within condition | NMES | WS vs SS | C-DLPFC | 1.456 | 0.176 | 0.4 | No |
| F2 | Task comparison within condition | NMES | WS vs SS | I-FEF | 0.121 | 0.905 | 0.905 | No |
| F2 | Task comparison within condition | NMES | WS vs SS | C-FEF | 1.032 | 0.314 | 0.502 | No |
| **F2** | **Task comparison within condition** | **NMES** | **WS vs SS** | **I-FPC** | **4.152** | **0.0001** | **0.002** | **Yes** |
| F2 | Task comparison within condition | NMES | WS vs SS | C-FPC | 1.855 | 0.069 | 0.276 | No |
| F3 | Task comparison within condition | No stimulation | WS vs SS | I-SMG | 0.853 | 0.398 | 0.490 | No |
| F3 | Task comparison within condition | No stimulation | WS vs SS | C-SMG | -1.590 | 0.119 | 0.339 | No |
| F3 | Task comparison within condition | No stimulation | WS vs SS | I-SMA/PMC | -1.367 | 0.175 | 0.38 | No |
| F3 | Task comparison within condition | No stimulation | WS vs SS | C-SMA/PMC | 0.513 | 0.609 | 0.650 | No |
| F3 | Task comparison within condition | No stimulation | WS vs SS | I-M1 | -0.897 | 0.391 | 0.490 | No |
| F3 | Task comparison within condition | No stimulation | WS vs SS | C-M1 | -1.09 | 0.301 | 0.482 | No |
| F3 | Task comparison within condition | No stimulation | WS vs SS | I-SAA | 1.568 | 0.127 | 0.339 | No |
| F3 | Task comparison within condition | No stimulation | WS vs SS | C-SAA | -0.909 | 0.37 | 0.490 | No |
| F3 | Task comparison within condition | No stimulation | WS vs SS | I-S1 | -1.203 | 0.238 | 0.423 | No |
| F3 | Task comparison within condition | No stimulation | WS vs SS | C-S1 | 1.580 | 0.124 | 0.339 | No |
| F3 | Task comparison within condition | No stimulation | WS vs SS | I-DLPFC | 0.614 | 0.553 | 0.632 | No |
| F3 | Task comparison within condition | No stimulation | WS vs SS | C-DLPFC | -0.267 | 0.795 | 0.795 | No |
| F3 | Task comparison within condition | No stimulation | WS vs SS | I-FEF | -2.173 | 0.041 | 0.219 | No |
| F3 | Task comparison within condition | No stimulation | WS vs SS | C-FEF | -1.353 | 0.19 | 0.38 | No |
| **F3** | **Task comparison within condition** | **No stimulation** | **WS vs SS** | **I-FPC** | **-3.440** | **0.001** | **0.018** | **Yes** |
| **F3** | **Task comparison within condition** | **No stimulation** | **WS vs SS** | **C-FPC** | **-3.108** | **0.003** | **0.024** | **Yes** |
| F4 | Stimulation comparison within task | WS | tDCS vs NMES | I-SMG | -1.477 | 0.147 | 0.548 | No |
| F4 | Stimulation comparison within task | WS | tDCS vs NMES | C-SMG | 0.942 | 0.351 | 0.562 | No |
| F4 | Stimulation comparison within task | WS | tDCS vs NMES | I-SMA/PMC | 0.156 | 0.876 | 0.883 | No |
| F4 | Stimulation comparison within task | WS | tDCS vs NMES | C-SMA/PMC | 0.295 | 0.768 | 0.883 | No |
| F4 | Stimulation comparison within task | WS | tDCS vs NMES | I-M1 | -1.238 | 0.244 | 0.548 | No |
| F4 | Stimulation comparison within task | WS | tDCS vs NMES | C-M1 | -0.244 | 0.812 | 0.883 | No |
| F4 | Stimulation comparison within task | WS | tDCS vs NMES | I-SAA | -0.148 | 0.883 | 0.883 | No |
| F4 | Stimulation comparison within task | WS | tDCS vs NMES | C-SAA | -3.026 | 0.005 | 0.078 | No |
| F4 | Stimulation comparison within task | WS | tDCS vs NMES | I-S1 | 0.424 | 0.674 | 0.883 | No |
| F4 | Stimulation comparison within task | WS | tDCS vs NMES | C-S1 | -1.469 | 0.152 | 0.548 | No |
| F4 | Stimulation comparison within task | WS | tDCS vs NMES | I-DLPFC | 0.988 | 0.347 | 0.562 | No |
| F4 | Stimulation comparison within task | WS | tDCS vs NMES | C-DLPFC | -1.451 | 0.177 | 0.548 | No |
| F4 | Stimulation comparison within task | WS | tDCS vs NMES | I-FEF | 0.321 | 0.751 | 0.883 | No |
| F4 | Stimulation comparison within task | WS | tDCS vs NMES | C-FEF | 1.123 | 0.274 | 0.548 | No |
| F4 | Stimulation comparison within task | WS | tDCS vs NMES | I-FPC | 1.298 | 0.2 | 0.548 | No |
| F4 | Stimulation comparison within task | WS | tDCS vs NMES | C-FPC | 1.250 | 0.217 | 0.548 | No |
| F5 | Stimulation comparison within task | WS | tDCS vs No stimulation | I-SMG | -1.954 | 0.057 | 0.216 | No |
| F5 | Stimulation comparison within task | WS | tDCS vs No stimulation | C-SMG | 0.545 | 0.589 | 0.724 | No |
| F5 | Stimulation comparison within task | WS | tDCS vs No stimulation | I-SMA/PMC | -1.082 | 0.282 | 0.489 | No |
| F5 | Stimulation comparison within task | WS | tDCS vs No stimulation | C-SMA/PMC | -0.707 | 0.481 | 0.641 | No |
| F5 | Stimulation comparison within task | WS | tDCS vs No stimulation | I-M1 | -1.503 | 0.164 | 0.375 | No |
| F5 | Stimulation comparison within task | WS | tDCS vs No stimulation | C-M1 | 1.035 | 0.325 | 0.489 | No |
| F5 | Stimulation comparison within task | WS | tDCS vs No stimulation | I-SAA | -1.76 | 0.088 | 0.235 | No |
| F5 | Stimulation comparison within task | WS | tDCS vs No stimulation | C-SAA | -1 | 0.325 | 0.489 | No |
| F5 | Stimulation comparison within task | WS | tDCS vs No stimulation | I-S1 | -0.154 | 0.878 | 0.902 | No |
| F5 | Stimulation comparison within task | WS | tDCS vs No stimulation | C-S1 | -1.892 | 0.068 | 0.216 | No |
| F5 | Stimulation comparison within task | WS | tDCS vs No stimulation | I-DLPFC | 0.257 | 0.802 | 0.902 | No |
| F5 | Stimulation comparison within task | WS | tDCS vs No stimulation | C-DLPFC | 0.127 | 0.902 | 0.902 | No |
| F5 | Stimulation comparison within task | WS | tDCS vs No stimulation | I-FEF | 2.028 | 0.055 | 0.216 | No |
| F5 | Stimulation comparison within task | WS | tDCS vs No stimulation | C-FEF | 0.985 | 0.336 | 0.489 | No |
| **F5** | **Stimulation comparison within task** | **WS** | **tDCS vs No stimulation** | **I-FPC** | **3.180** | **0.002** | **0.020** | **Yes** |
| **F5** | **Stimulation comparison within task** | **WS** | **tDCS vs No stimulation** | **C-FPC** | **4.086** | **0.0001** | **0.002** | **Yes** |
| F6 | Stimulation comparison within task | WS | NMES vs No stimulation | I-SMG | -0.721 | 0.475 | 0.610 | No |
| F6 | Stimulation comparison within task | WS | NMES vs No stimulation | C-SMG | 0.043 | 0.966 | 0.966 | No |
| F6 | Stimulation comparison within task | WS | NMES vs No stimulation | I-SMA/PMC | -1.137 | 0.259 | 0.576 | No |
| F6 | Stimulation comparison within task | WS | NMES vs No stimulation | C-SMA/PMC | -0.854 | 0.395 | 0.576 | No |
| F6 | Stimulation comparison within task | WS | NMES vs No stimulation | I-M1 | 0.204 | 0.842 | 0.911 | No |
| F6 | Stimulation comparison within task | WS | NMES vs No stimulation | C-M1 | 0.984 | 0.348 | 0.576 | No |
| F6 | Stimulation comparison within task | WS | NMES vs No stimulation | I-SAA | -1.508 | 0.141 | 0.541 | No |
| F6 | Stimulation comparison within task | WS | NMES vs No stimulation | C-SAA | 1.366 | 0.181 | 0.541 | No |
| F6 | Stimulation comparison within task | WS | NMES vs No stimulation | I-S1 | -0.688 | 0.496 | 0.610 | No |
| F6 | Stimulation comparison within task | WS | NMES vs No stimulation | C-S1 | -1.415 | 0.167 | 0.541 | No |
| F6 | Stimulation comparison within task | WS | NMES vs No stimulation | I-DLPFC | -0.888 | 0.396 | 0.576 | No |
| F6 | Stimulation comparison within task | WS | NMES vs No stimulation | C-DLPFC | 1.096 | 0.299 | 0.576 | No |
| F6 | Stimulation comparison within task | WS | NMES vs No stimulation | I-FEF | 1.313 | 0.203 | 0.541 | No |
| F6 | Stimulation comparison within task | WS | NMES vs No stimulation | C-FEF | 0.187 | 0.854 | 0.911 | No |
| F6 | Stimulation comparison within task | WS | NMES vs No stimulation | I-FPC | 2.034 | 0.047 | 0.375 | No |
| F6 | Stimulation comparison within task | WS | NMES vs No stimulation | C-FPC | 2.887 | 0.006 | 0.089 | No |
| F7 | Stimulation comparison within task | SS | tDCS vs NMES | I-SMG | 1.479 | 0.146 | 0.464 | No |
| F7 | Stimulation comparison within task | SS | tDCS vs NMES | C-SMG | 0.652 | 0.518 | 0.637 | No |
| **F7** | **Stimulation comparison within task** | **SS** | **tDCS vs NMES** | **I-SMA/PMC** | **3.43** | **0.0009** | **0.014** | **Yes** |
| F7 | Stimulation comparison within task | SS | tDCS vs NMES | C-SMA/PMC | 1.169 | 0.245 | 0.464 | No |
| F7 | Stimulation comparison within task | SS | tDCS vs NMES | I-M1 | -0.105 | 0.919 | 0.980 | No |
| F7 | Stimulation comparison within task | SS | tDCS vs NMES | C-M1 | 1.342 | 0.209 | 0.464 | No |
| F7 | Stimulation comparison within task | SS | tDCS vs NMES | I-SAA | 1.465 | 0.153 | 0.464 | No |
| F7 | Stimulation comparison within task | SS | tDCS vs NMES | C-SAA | 0.791 | 0.435 | 0.624 | No |
| F7 | Stimulation comparison within task | SS | tDCS vs NMES | I-S1 | 1.143 | 0.261 | 0.464 | No |
| F7 | Stimulation comparison within task | SS | tDCS vs NMES | C-S1 | 1.792 | 0.083 | 0.440 | No |
| F7 | Stimulation comparison within task | SS | tDCS vs NMES | I-DLPFC | 1.028 | 0.328 | 0.5248 | No |
| F7 | Stimulation comparison within task | SS | tDCS vs NMES | C-DLPFC | 1.281 | 0.229 | 0.464 | No |
| F7 | Stimulation comparison within task | SS | tDCS vs NMES | I-FEF | -0.219 | 0.829 | 0.947 | No |
| F7 | Stimulation comparison within task | SS | tDCS vs NMES | C-FEF | 0.739 | 0.468 | 0.624 | No |
| F7 | Stimulation comparison within task | SS | tDCS vs NMES | I-FPC | 2.825 | 0.007 | 0.053 | No |
| F7 | Stimulation comparison within task | SS | tDCS vs NMES | C-FPC | 0.021 | 0.983 | 0.983 | No |
| F8 | Stimulation comparison within task | SS | tDCS vs No stimulation | I-SMG | 0.352 | 0.727 | 0.966 | No |
| F8 | Stimulation comparison within task | SS | tDCS vs No stimulation | C-SMG | -1.596 | 0.118 | 0.471 | No |
| F8 | Stimulation comparison within task | SS | tDCS vs No stimulation | I-SMA/PMC | 0.818 | 0.415 | 0.8544 | No |
| F8 | Stimulation comparison within task | SS | tDCS vs No stimulation | C-SMA/PMC | -0.667 | 0.506 | 0.8544 | No |
| F8 | Stimulation comparison within task | SS | tDCS vs No stimulation | I-M1 | -0.201 | 0.845 | 0.966 | No |
| F8 | Stimulation comparison within task | SS | tDCS vs No stimulation | C-M1 | 0.67 | 0.518 | 0.854 | No |
| F8 | Stimulation comparison within task | SS | tDCS vs No stimulation | I-SAA | 1.319 | 0.196 | 0.523 | No |
| F8 | Stimulation comparison within task | SS | tDCS vs No stimulation | C-SAA | -0.077 | 0.939 | 0.996 | No |
| F8 | Stimulation comparison within task | SS | tDCS vs No stimulation | I-S1 | -0.006 | 0.996 | 0.996 | No |
| F8 | Stimulation comparison within task | SS | tDCS vs No stimulation | C-S1 | -0.284 | 0.778 | 0.966 | No |
| F8 | Stimulation comparison within task | SS | tDCS vs No stimulation | I-DLPFC | 0.216 | 0.833 | 0.966 | No |
| F8 | Stimulation comparison within task | SS | tDCS vs No stimulation | C-DLPFC | 0.643 | 0.534 | 0.8544 | No |
| F8 | Stimulation comparison within task | SS | tDCS vs No stimulation | I-FEF | -1.347 | 0.192 | 0.523 | No |
| **F8** | **Stimulation comparison within task** | **SS** | **tDCS vs No stimulation** | **C-FEF** | **-3.139** | **0.005** | **0.026** | **Yes** |
| **F8** | **Stimulation comparison within task** | **SS** | **tDCS vs No stimulation** | **I-FPC** | **-4.013** | **0.0002** | **0.001** | **Yes** |
| **F8** | **Stimulation comparison within task** | **SS** | **tDCS vs No stimulation** | **C-FPC** | **-4.026** | **0.0002** | **0.001** | **Yes** |
| F9 | Stimulation comparison within task | SS | NMES vs No stimulation | I-SMG | -1.091 | 0.281 | 0.409 | No |
| F9 | Stimulation comparison within task | SS | NMES vs No stimulation | C-SMG | -2.085 | 0.0430 | 0.119 | No |
| **F9** | **Stimulation comparison within task** | **SS** | **NMES vs No stimulation** | **I-SMA/PMC** | **-3.094** | **0.003** | **0.0138** | **Yes** |
| F9 | Stimulation comparison within task | SS | NMES vs No stimulation | C-SMA/PMC | -1.576 | 0.118 | 0.235 | No |
| F9 | Stimulation comparison within task | SS | NMES vs No stimulation | I-M1 | -0.162 | 0.875 | 0.883 | No |
| F9 | Stimulation comparison within task | SS | NMES vs No stimulation | C-M1 | -1.294 | 0.225 | 0.36 | No |
| F9 | Stimulation comparison within task | SS | NMES vs No stimulation | I-SAA | -0.36 | 0.721 | 0.824 | No |
| F9 | Stimulation comparison within task | SS | NMES vs No stimulation | C-SAA | -0.877 | 0.387 | 0.516 | No |
| F9 | Stimulation comparison within task | SS | NMES vs No stimulation | I-S1 | -2.089 | 0.045 | 0.1192 | No |
| F9 | Stimulation comparison within task | SS | NMES vs No stimulation | C-S1 | -1.774 | 0.086 | 0.196 | No |
| F9 | Stimulation comparison within task | SS | NMES vs No stimulation | I-DLPFC | -0.64 | 0.536 | 0.660 | No |
| F9 | Stimulation comparison within task | SS | NMES vs No stimulation | C-DLPFC | -0.15 | 0.883 | 0.883 | No |
| F9 | Stimulation comparison within task | SS | NMES vs No stimulation | I-FEF | -1.568 | 0.132 | 0.235 | No |
| **F9** | **Stimulation comparison within task** | **SS** | **NMES vs No stimulation** | **C-FEF** | **-3.137** | **0.005** | **0.020** | **Yes** |
| **F9** | **Stimulation comparison within task** | **SS** | **NMES vs No stimulation** | **I-FPC** | **-4.741** | **0.00002** | **0.0003** | **Yes** |
| **F9** | **Stimulation comparison within task** | **SS** | **NMES vs No stimulation** | **C-FPC** | **-3.967** | **0.0002** | **0.002** | **Yes** |

Note: Exploratory paired t-tests were organized into nine comparison families. Benjamini–Hochberg false discovery rate (BH-FDR) correction was applied separately across the 16 ROIs within each comparison family. Families F1–F3 compared water swallowing (WS) and saliva swallowing (SS) within each stimulation condition. Families F4–F9 compared stimulation conditions within each swallowing task. Statistical significance after correction was defined as q < 0.05. ROI labels follow the lesion-side normalization used in the manuscript, where I denotes ipsilesional and C denotes contralesional regions.
