# Supplementary material for: Task-dependent cortical responses to peripheral and central neuromodulation during water and saliva swallowing after stroke: an exploratory fNIRS pilot study
Source: Front Hum Neurosci. 2026 Aug 24;20:1907700. doi: 10.3389/fnhum.2026.1907700 (PMC13547267; doi:10.3389/fnhum.2026.1907700)
Supplement: Supplementary file 2 [file Table_2.docx]

## **Supplementary Table S2. Clinical Outcome Verification Summary**

| **Outcome** | **Condition** | **Improved** | **Unchanged** | **Worsened** | **Z** | **Exact p** | **r** | **Median change** | **Bootstrap 95% CI** |
| --- | --- | --- | --- | --- | --- | --- | --- | --- | --- |
| KWDT | No stimulation | 0/11 | 11/11 | 0/11 | 0.000 | 1.0000 | 0.000 | 0 | [0, 0] |
| KWDT | NMES | 4/11 | 7/11 | 0/11 | −1.643 | 0.1250 | 0.495 | 0 | [−1, 0] |
| KWDT | tDCS | 4/11 | 7/11 | 0/11 | −1.643 | 0.1250 | 0.495 | 0 | [−1, 0] |
| FOIS | No stimulation | 0/11 | 11/11 | 0/11 | 0.000 | 1.0000 | 0.000 | 0 | [0, 0] |
| FOIS | NMES | 4/11 | 7/11 | 0/11 | −1.643 | 0.1250 | 0.495 | 0 | [0, 1] |
| FOIS | tDCS | 5/11 | 6/11 | 0/11 | −1.888 | 0.0625 | 0.569 | 0 | [0, 1] |

Note. Exact p values are two-sided. Wilcoxon effect size was calculated as r = |Z|/√N. Median change and bootstrap 95% CI refer to the signed post-minus-pre difference (10,000 resamples), not to the effect size r. For KWDT, negative change indicates improvement; for FOIS, positive change indicates improvement.
